# Sparse representations of high dimensional neural data

**DOI:** 10.1038/s41598-022-10459-7

**Published:** 2022-05-04

**Authors:** Sandeep K. Mody, Govindan Rangarajan

**Affiliations:** 1 Department of Mathematics, Indian Institute of Science, Bangalore, India; 2 Centre for Neuroscience, Indian Institute of Science, Bangalore, India

**Keywords:** Neuroscience, Computational neuroscience, Emotion, Computational neuroscience, Statistical methods

## Abstract

Conventional Vector Autoregressive (VAR) modelling methods applied to high dimensional neural time series data result in noisy solutions that are dense or have a large number of spurious coefficients. This reduces the speed and accuracy of auxiliary computations downstream and inflates the time required to compute functional connectivity networks by a factor that is at least inversely proportional to the true network density. As these noisy solutions have distorted coefficients, thresholding them as per some criterion, statistical or otherwise, does not alleviate the problem. Thus obtaining a sparse representation of such data is important since it provides an efficient representation of the data and facilitates its further analysis. We propose a fast Sparse Vector Autoregressive Greedy Search (SVARGS) method that works well for high dimensional data, even when the number of time points is relatively low, by incorporating only statistically significant coefficients. In numerical experiments, our methods show high accuracy in recovering the true sparse model. The relative absence of spurious coefficients permits accurate, stable and fast evaluation of derived quantities such as power spectrum, coherence and Granger causality. Consequently, sparse functional connectivity networks can be computed, in a reasonable time, from data comprising tens of thousands of channels/voxels. This enables a much higher resolution analysis of functional connectivity patterns and community structures in such large networks than is possible using existing time series methods. We apply our method to EEG data where computed network measures and community structures are used to distinguish emotional states as well as to ADHD fMRI data where it is used to distinguish children with ADHD from typically developing children.

## Introduction

Analysis of high dimensional data is of great current interest^1–5^. Examples of high dimensional data include high-throughput genomics^6^, economic and financial time series data^7^ and EEG data (with hundreds of channels). Another example, and the one that we are primarily interested in, is fMRI data^8^ with millions of voxels and hundreds of Regions of Interest (ROI’s). A popular mathematical model for representing such time series data is the Vector Autoregressive (VAR) model^9,10^. Though the VAR model by itself can be interesting, it is primarily used as a means to derive other useful quantities such as high-resolution power spectra, coherence, causality measures etc.^10,11^. In recent years, VAR models have been widely used (especially in the context of neuroscience) to obtain functional/effective connectivity networks through Granger causality^12–16^.

In many cases high dimensional data admit a sparse representation, for example from a generic geometric point of view^17^ or via signal component sparsity^18^ or via feature reduction^19^. Hence, when modelling such data using VAR models, it is important that these models are sparse. Sparse VAR models are those where the number of non-zero coefficients in the model is far fewer than the maximum possible number of coefficients. There are several additional reasons for using sparse VAR models. When applied to high dimensional neural data, traditional modelling methods yield dense and noisy VAR models where most of the coefficients are not statistically significant. Applying a fixed thresholding on the coefficients after model generation does not help since it leads to misidentification of interactions between the channels/voxels/ROIs. Further, it is computationally expensive and often numerically unstable to derive auxiliary quantities such as the power spectrum, coherence and causality measures from dense VAR models. More importantly, these quantities are corrupted by the presence of a large number of statistically insignificant coefficients in a dense model. Obtaining a sparse representation may be important, even in cases where the underlying system is not sparse, if we are interested only in the most significant interactions that adequately explain the observed data. Finally, one of the important motivations for representing high dimensional neural data using sparse VAR models arises from the fact that one can generate sparse functional connectivity network models from such VAR models (as described later in the paper).

Sparsity in networks corresponds to the situation where the actual number of edges in the network is much smaller than the maximum number of edges that is theoretically possible. Since actual networks that are large also tend to be sparse^20–22^, obtaining a sparse representation of the network from high dimensional data becomes critical. In the context of neuronal networks, a primary reason for sparsity is the well-known fact^23,24^ that more than 50% of brain’s energy consumption is required for signalling in such networks and for the use and maintenance of neurons. Such a high energy cost forces the wiring scheme in the network to be efficient^23,24^. One such efficient wiring scheme is a sparse network. In particular, for the human brain, the estimated numbers of neurons (*k*) is $8.6 \times 10^{10}$^25^ so that the maximum potential number of edges is approximately $10^{21}$. On the other hand, the actual number of edges is bounded above by the number of synapses which is estimated to be only $4 \times 10^{14}$. Even in other large networks, similar considerations of energy and other costs can lead to sparsity.

We address a contervailing point of view that suggests that anatomical networks are highly dense^26,27^. We note that in both the above works, the parcellation used resulted in 25 and 29 brain regions respectively and the dense network referred to is between these relatively coarse parcels. This density is not so surprising given that the activity at each of the individual ROI’s is an aggregate of the activity of a presumably much larger number of neuronal units within that ROI. In other words, merging “units” of activity will reduce the sparsity of a network and the greater the region of the averaging the more dense the network will appear. Thus, at least in theory, we posit that for a sufficiently high spatial resolution brain networks *will* be sparse. In particular Modha et al.^28^ discusses how small-worldness of the brain network contributes to this sparsity. We must admit here that in our second application to the DEAP EEG emotion dataset the parcellation implicit in our choice of channels is indeed very coarse. However in that case we appeal to the second leg of our sparsity argument—which is that we are interested only in the most significant interactions of the system that adequately explain the observed data. We note here that Gămănuţ et al.^26^ states that the observed log-normal distribution of connection weights between each of their ROI’s spans 5 orders of magnitude. This suggests that, in their case, there was sufficient variation in the weights to allow for extracting a sparse subset.

The method described in our paper is not directly applicable to vast collections of neurons but rather to smaller (yet large) networks, such as fMRI recordings made at the voxel level. These are networks whose nodes comprise neuronal ensembles contained in each voxel. There is evidence that such networks also, are sparse. This sparsity has been observed by Modha et al.^28^ using the anatomical and connectivity data in the CoCoMac^29^ (also see Supplementary Section 1) database of the Macaque brain.

Based on the above argument, for the analysis of brain signals to determine functional connectivity, we take the sparsity of the resulting network to be an a-priori requirement. The task then is to eliminate insignificant interactions and estimate the strength of the true interactions in the system. In the context of VAR modelling, this implies describing the data by means of a sparse VAR model. However, the standard methods for computing VAR models yield dense models and also require a very large number of time points (typically exceeding the number of lagged variables by at least an order of magnitude or more).

A well known penalized regression method for overcoming the problems of standard regression and obtaining sparsity is Lasso (Least Absolute Selection and Shrinkage Operator) Regression^30^. Another method for obtaining sparsity is to threshold the standard VAR solution either at the coefficient level or at the Granger Causality level using an appropriate statistical procedure. However these penalized methods yield semi-sparse models and this makes computation of Granger Causality or Conditional Granger Causality computationally very expensive. A comparison of the accuracy and computational speed of Lasso as implemented in the glmnet package^31^ with SVARGS is included in section “Computational issues”. On the other hand thresholding dense models leads to misidentification of interactions between variables/channels since it thresholds values that are already distorted.

Given the inadequacies of the existing approaches, in order to obtain highly sparse VAR models and the corresponding sparse networks, we propose a bottom up, greedy algorithm (Sparse Vector Auto-Regressive Greedy Search—SVARGS). In this method, testing for statistical significance of coefficients, and final choice of model using an information criterion, is built into the model generation algorithm. Using simulated data generated from large random sparse VAR models, we test the performance our methods for different lengths of data.

We also compute the Conditional Granger Causality (CGC)^32,33^ values from the models obtained for the simulated data. Suppose we have a system consisting of a collection of time series variables $W_{t} = {X_{t}, Y_{t}, Z_{t}}$ (where each of $X_{t}$, $Y_{t}$ and $Z_{t}$ may themselves be collections of variables). Denote the past of $X_{t}$ by $X_{t-}$ and and similarly for $Y_{t}$, $Z_{t}$ and $W_{t}$. The Granger Causality $F_{Y \rightarrow X}$ from *Y* to *X* conditioned on the past of the variables $Z_{t}$ is defined by:

1 $$\begin{aligned} F_{Y \rightarrow X} = \log \frac{L(X_{t} | W_{t-})}{L(X_{t} | X_{t-}, Z_{t-})} \end{aligned}$$

where $L(X_{t} | W_{t-} )$ is the likelihood of $X_{t}$ given the past of $W_{t}$ and $L(X_{t} | X_{t-}, Z_{t-})$ is the likelihood of $X_{t}$ given the past of $X_{t}, Z_{t}$.

CGC is superior to pairwise Granger causality since it eliminates the influence of the intermediate variables $Z_{t}$. In our analysis, CGCs play an important role since they are used to construct functional connectivity networks representing the causal interaction between different variables in the system.

We use our methods to analyse experimental data from the public domain. In the first application, preprocessed, pre-parcelled, multi-subject resting state data containing 954 clusters from the ADHD-200 project is analysed using the SVARGS method. Using the functional connectivity network measures obtained from the VAR model of this data, we are able to distinguish between ADHD and typically developing children using global and local network measures. In the second application multi-subject EEG data from the DEAP emotion database is analysed using our method. The resulting networks are used as features to classify subject assessed emotional states as high intensity or low intensity.

## Methods

We start with high dimensional data $\hat{Y}$, for example an EEG recording with a large number of channels or an fMRI data set with a large number of voxels or ROI’s, recorded at discrete time intervals. The data can have more than one trial. Our implementations work transparently regardless of the number of trials in the data set.

First we fit a sparse VAR model to this data. Next we determine the network of interactions between the channels/voxels/ROIs by computing Conditional Granger Causality (CGC) strengths^11,32,33^ from the fitted VAR model. The sparse VAR model allows efficient computation of interactions between single variables as well as between any variable blocks of interest. The sparse VAR model can also be used to compute other quantities of interest such as coherence, power spectrum etc.

We begin with the assumption that the observed data $\hat{Y}$ can be modelled by a vector of variables $Y_{t} = \{y_{t}^{(1)}, y_{t}^{(2)}, \ldots , y_{t}^{(k)}\}^{T}$ representing a multivariate Vector Auto Regressive (VAR) stochastic process:

2 $$\begin{aligned} Y_{t} = A_{1}Y_{t-1} + A_{2}Y_{t-2} + \ldots , A_{p}Y_{t-p} + \varepsilon _{t} \end{aligned}$$

We also assume that the process is weakly stationary, by which we imply that the mean vector of $Y_{t}$ is independent of the time *t*, and for any times *t* and *s*, the covariance matrix $Cov(Y_{t},Y_{s})$ depends only on $t-s$.In addition we assume that the process has zero mean (since any systematic trend can be always removed from the data). The equation above expresses the (column) vector *Y* at time *t* as a linear function of its delayed (lagged) values. The symbols $A_{1}, A_{2}, \ldots A_{p}$ are $k \times k$ real coefficient matrices and *p* is the maximum delay (the order) of the model. The variable $\varepsilon _{t}$ models the residuals and we assume that $\varepsilon _{t}$ is normally distributed white noise with constant covariance matrix:

3 $$\begin{aligned} \Sigma _{\varepsilon } = E(\varepsilon _{t}\varepsilon _{t}^{T}) \end{aligned}$$

For such a process, the likelihood of the observed values of any variable is proportional to the reciprocal of the determinants of the error covariance matrices of that variable involved (see^34^, page 348, eq. 5.4.9). Using Eq. (1), this enables us to estimate the Granger Causality strengths for the time series variables by estimating the the covariance matrices of the different variables involved (see Supplementary Section 3.4.1 for details). Moreover, there exists a spectral representation of the conditional Granger Causality where the sum of the CGC between any two variables over different frequencies is equal to the total CGC strength (see^32^). The formulas that result are presented in Supplementary Section 3.4.2. See also^11^.

The standard way to estimate the matrices $A_{1}, A_{2}, \ldots A_{p}$ as well as the error covariance $\Sigma _{\varepsilon }$, is to use either linear regression or, more or less equivalently, the Yule–Walker equations. These standard methods are described in Supplementary Section 2.1. Our objective, however, is to estimate highly *sparse* matrices $A_{1}, A_{2}, \ldots A_{p}$ such that Eq. (2) and Eq. (3) best represent the observed time series data and the procedure for this is described in the following section.

### SVARGS method

We propose a greedy method, SVARGS (Sparse VAR Greedy Search), to overcome the drawbacks of standard vector auto-regression estimation techniques for sparse models. The SVARGS method is a variation on the technique described in^35^, and it was implemented earlier for a single variable case in a different context^36^.

SVARGS is based on comparing the relative likelihoods of candidate solutions. Instead of including, at one go, all of variables at all possible lags as predictors for each of the response variables, each variable (block) at each lag is included or removed as a predictor (block), one at a time, based on the size of the observed error in the response variable (block) before and after the inclusion or removal. The algorithm requires that we be able to determine whether an added coefficient is significant. The technical procedure for accomplishing this is described in Supplementary Section 2.3.1 and Supplementary Section 2.3.4.

Let $T = \{t_{1}, t_{2}, \dots , t_{q}\}$ denote the set of potential lags. Typically $T = \{1,2,\ldots , p\}$ where *p* is the maximum model order permitted. We assume that, for all times *t*, the set of atomic variables $\{y_{t}^{(1)}, y_{t}^{(2)}, \ldots , y_{t}^{(k)}\}$ is partitioned into *J* disjoint blocks $\varvec{r}_{t}^{(1)}, \varvec{r}_{t}^{(2)}, \ldots \varvec{r}_{t}^{(J)}$. Here each block $\varvec{r}_{t}^{(j)}, j \in 1,2 \ldots J$ consists of some ordered subset (i.e.: vector) of atomic variables $\{y_{t}^{(i)}: i \in K_{j}\}$ where the integer subsets $K_{j} \subset \{1,2, \ldots k\}, j \in {1,2, \ldots J}$ are disjoint and $\cup _{j=1}^{J}K_{j} = \{1,2, \ldots k\}$. Let ${\mathbb {X}}$ denote the set of all lagged variable vectors $\{\varvec{r}_{t}^{(j)}: j \in \{1,2, \ldots , J\}, t \in T\}$, which form the full set of potential block predictors (regressors) for the fit. In terms of these block variables the VAR equation (2) for the *j*th vector block can be rewritten as (with a possible change of ordering of the variables $y_{t}^{(1)}, y_{t}^{(2)}, \ldots , y_{t}^{(k)}$):

4 $$\begin{aligned} \varvec{r}_{t}^{(j)} = \sum _{i \in T}\left[ A_{i}^{(K_j,K_{1})}\varvec{r}_{t-i}^{(1)} \;+\; A_{i}^{(K_j,K_{2})}\varvec{r}_{t-i}^{(2)} \;+\; \ldots \;+\; A_{i}^{(K_j,K_{j})}\varvec{r}_{t-i}^{(J)}\right] \quad + \quad \varepsilon _{t}^{(K_{j})} \end{aligned}$$

where $A_{i}^{(K_j, K_{m})}$ is the submatrix of $A_{i}$ formed by extracting those rows corresponding to the indices in block $K_{j}$ and those columns corresponding to the indices in block $K_{m}$.

We shall denote by $VAR^{(j)}$ the formal equation (4) above. In Eq. (4), those predictor blocks corresponding to any zero coefficient blocks may be elided from the expression. We shall refer to the elided equation for the *j*th block as the *model* $M^{(j)}$. To start the algorithm, initialize the block number *j* to 1 and the model $M^{(1)}$ for this first block of variables to the trivial VAR model:

5 $$\begin{aligned} \varvec{r}_{t}^{(1)} = \varepsilon _{t}^{(K_{1})} \quad \text{(Initial } \text{ trivial } \text{ model } \text{ for } \text{ block } \text{ j } \text{= } \text{1) } \end{aligned}$$

#### SVARGS algorithm

1. Let $VAR_{j}$ denote the group of VAR equations for the *j*th variable block. Let $M^{(j)}$ be the current model for this block of variables. Initialize a value $p_{entry}$ to a small pre-selected value $p_{min}$.
2. Compute the error covariance matrix $\Sigma _{\varepsilon }$ of $M^{(j)}$.
3. For each lagged regressor block $x \in {\mathbb {X}}$ not already present in the RHS of $M^{(j)}$:
  - Let $M^{+}_{j}$ denote the augmented block of equations obtained by including the regressors in *x* on the RHS of each of the constituent equations of $M^{(j)}$. Use OLS to fit $M^{+}_{j}$ to the observed data.
  - Compute the error covariance matrix $\Sigma _{\varepsilon }^{+}$ of $M^{+}_{j}$. Record the ratio $det(\Sigma _{\varepsilon })/det(\Sigma _{\varepsilon }^{+})$ as well as the regressor block and coefficient matrix corresponding to this ratio.
4. From step 3, find the regressor block $x^{+}$ and associated coefficient matrix $C^{+}$ that gives the largest determinant ratio. This largest determinant ratio is our entry test statistic.
5. Now test if the entry test statistic is greater than unity.
6. If the entry test statistic has p-value less than or equal to $p_{entry}$, update the set of equations in $M^{(j)}$ by adding $C^{+}x^{+}$ to its RHS. Let $\Sigma _{\varepsilon }$ be the error covariance matrix for this, possibly new, current model $M^{(j)}$.
7. Next, for each lagged regressor block *x* present in the RHS of $M^{(j)}$:
  - let $M^{-}_{j}$ denote the contracted equations obtained by removing all the regressors in *x* from the RHS of $M^{(j)}$. Use OLS to fit $M^{-}_{j}$ to the observed data.
  - Compute the error covariance matrix $\Sigma _{\varepsilon }$ of $M^{-}_{j}$. Record the ratio $det(\Sigma _{\varepsilon }^{-})/det(\Sigma _{\varepsilon })$ as well as the regressor block corresponding to this ratio.
8. From step 7, find the regressor block $x^{-}$ that gives the smallest determinant ratio. This smallest determinant ratio is our exit test statistic.
9. Test if the exit test statistic is greater than unity.
10. Let $p_{exit} = 2p_{entry}$. If the exit test statistic has p-value greater than $p_{exit}$, *remove* the regressor block $x^{-}$ from the set of equations $M^{(j)}$. These, possibly updated, equations again become the current model.
11. Record $M^{(j)}$ and its corresponding fit quality using the *extended SBC* criterion.
12. If any coefficient has been added or removed, return to step 2. Otherwise if $p_{entry} < p_{max}$, increment $p_{entry}$ using a small multiplicative factor and return to step 2.
13. Based on the recorded models (step 11) and the corresponding recorded fit qualities, set $M^{(j)}$ to the model having the best fit quality.
14. If *j* is less than *J*, increment *j* and return to step 1.

In the algorithm above, the values $p_{min}$ and $p_{max}$ must be chosen a-priori. During the course of the fit, $p_{entry}$ is progressively increased until it equals or exceeds the chosen $p_{max}$. The exit p-value $p_{exit}$ must always be greater than $p_{entry}$. In all the computations presented here we chose $p_{exit} = 2p_{entry}$. However numerical experiments showed that varying the ratio $p_{exit}/p_{entry}$ somewhat, makes little difference to the results produced by the algorithm. Guidance on how to choose the values $p_{min}$ and $p_{max}$ and increment $p_{entry}$ is provided in Supplementary Section 2.3.6.

The log-ratio of the determinant of the error covariance’s of a component block *r* before and after entry of a component block *s* at some lag $\tau$ is a measure of the conditional Granger Causality strength from component *s* to component *r* at lag $\tau$ (given the existing set of dependencies as expressed by the current values of the model coefficients). The procedure for testing this Granger Causality statistic is discussed in Supplementary Section 2.3. If the blocks are atomic (that is have only one variable), then this Granger Causality statistic is proportional to the *F*-statistic in linear regression.

To summarize the algorithm, starting from a suitable initial VAR model (typically the trivial model), interactions between lagged variable blocks are added or removed depending on whether the interaction significantly increases the likelihood that model explains the data. For every model update, the coefficient locations are recorded. If no further interactions can be added or removed, the $p_{entry}$ value is increased slightly. This process is continued until $p_{entry}$ reached a pre-specified maximum value. The optimal solution is chosen from the set of solutions obtained by using the *extended SBC* information criterion^37^. See Supplementary Section 2.3.7 for a description of extended SBC.

We emphasise here that our method may not be suitable for cases where the true model is genuinely dense (except perhaps for very small systems). The limitations of the method and the practical meaning of the sparsity assumption are discussed in section “Discussion and conclusions”.

Source code for the algorithm presented as well as the url required to access updated versions of the code is included in the supplementary materials.

### Computational issues

In implementing the above algorithm, the following non-trivial computational issues were addressed. (1) Fast computation of pairwise and conditional Granger causality from large VAR models. (2) Fast testing of nested models during SVARGS fit. These are discussed in Supplementary Section 5 and Supplementary Section 6.

### A classification strategy

Later we apply our method to experimental data. In our first application, we analyse multi-subject EEG data from the DEAP emotion database (section “Analysis of DEAP emotion dataset”). Network measures based on the resulting networks are used as features to classify subject assessed emotional states as high intensity or low intensity. In the second application, we use the same methods on fMRI data from the ADHD-200 dataset to distinguish and classify fMRI scans of ADHD and typically developing children and (section “Application to ADHD fMRI data”). Both applications follow the same basic procedure: Apply the SVARGS method to compute a VAR model from the time-series data for each data instance (subject/video). Then compute the Conditional GC matrix for each data instance from the VAR model for that instance as described in Supplementary Section 3 and Supplementary Section 3.4. As mentioned earlier, this CGC matrix then becomes the weighted adjacency matrix for the functional connectivity network. No arbitrary thresholding is required as the CGC matrix is already sparse due to the sparsity of the underlying VAR model. Finally compute various network measures based on the connectivity matrix—these measures can be used to distinguish or classify the phenotypes of interest in the dataset. In the case of the two datasets above, we used the network measures listed in Table 1. In the following sections, we will refer to this table when required. See^38^ for an explanation of these measures.

**Table 1:** Network measures computed. See^38^ for an explanation of these measures.

Measure type	Measure
Scalar	(1) Edge Count, (2) Matrix Density, (3) Characteristic Path Length, (4) Global Efficiency, (5) Transitivity, (6) Assortativity-in-in, (7) Assortativity-in-out, (8) Assortativity-out-in, (9) Assortativity-out-out
Node-wise weighted	(1) Strengths-in, (2) Strengths-out, (3) Strengths-total, (4) Local Efficiencies, (5) Participation-in, (6) Participation-out, (7) Clustering Coefficients, (8) Closeness Centralities-in, (9) Closeness Centralities-out, (10) Closeness Centralities-total, (11) Eigenvector Centralities-in, (12) Eigenvector Centralities-out, (13) Sub-graph Centralities, (14) Node-Betweenness Centralities
Binary weighted	(1) Degrees-in, (2) Degrees-out, (3) Degrees-total, (4) Flow Coeffs, (5) Flow Path Counts, (6) Coreness Centralities

Our goal is to classify brain states using adaptive boosting (Adaboost:^39^) methods. For the basic tree classifier, we use the fitctree() Matlab function which implements the CART algorithm^40^, a variation of the C4.5 algorithm^41^. There are several parameters that may be supplied to the fitctree() function. All of them were left at their Matlab defaults, except:

1. The spitting criterion: We use the information-gain.
2. Feature selection criterion for splitting: We use the curvature test. The curvature test selects the split predictor that minimizes the p-value of chi-square tests of independence between each feature and the response^42,43^.
3. Tree pruning: We omit pruning of the individual trees in the ensemble.

Using the tree classifier above as the base classifier, we use the adaboost-SAMME^44^ classification algorithm to construct the ensemble of tree learners. For any given adaboost round, each of the learners used a randomly chosen subset of features of size approximately the square root of the number of available features.

### Feature preselection

Some of the measures in Table 1 and some of the nodes/channels had lesser explanatory power than others. We used an in-house measure and node pruning technique to select the most discriminatory measures and nodes. The main idea behind this technique is to use the *information gain* (or entropy decrease), referred to in short as the *gain*, as way to select those measures that are most relevant to the classifier. In short, the gain at any tree splitting step is the difference in the entropies of the data partition before and after the split. In any single tree classification, we define the gain of a single feature *f* as simply the sum of all the gain values computed every time a tree split occurs on the feature *f*. As the Adaboost algorithm involves an ensemble of trees, the gain of any feature is the *learner-weight* weighted sum of the set of gains of that feature from the trees in the ensemble. The cumulative gain of any given measure in an adaboost run is then the sum of the individual feature gains in the block of features associated with that measure. The calculation of these gains does not involve significant additional computation as they are calculated as part of the decision tree algorithm during a classification run. The measure selection is then done by separating those measures that have high gain from those that have low gain. The Supplementary Section 7 describes the algorithm used to achieve this separation. The same procedure was also also used for node selection (ie: selection of blocks of features corresponding to individual nodes (channels)). This further improved the classification accuracy. For the ADHD data, see also section “ADHD feature preselection and classification” and for DEAP emotion data see also Supplementary Section 8.2.

## Results of numerical simulations

Our *SVARGS* method as well as the Lasso regression method as implemented in the glmenet package (see^45^ for the glmnet matlab package and^46^ for the documentation) were both tested on simulated data. We generated sparse stable models of 4 different sizes and generated simulated data from these. The model sizes, k (number of response variables) chosen were $k = 35$ with densities between $1.2\%$ and $2.4\%$, $k = 100$ with densities between $0.4\%$ and $0.9\%$, $k = 300$ with densities between $0.13\%$ and $0.25\%$, and $k = 500$ with densities between $0.08\%$ and $0.18\%$. With these densities, for each model size and data length, the average number of coefficients per equation was between 3 and 8 and also less than a twentieth of the number of time points. The simulated data lengths, N (number of time points) were $N = 150, N = 200, N = 250, N = 300, N = 350, N = 400$ and $N = 450$. For each model size and each data length 50 random “true” stable sparse models were generated, yielding a total of 1400 VAR models. SVARGS was then tested by running it on each of the resulting 1400 data sets. In all cases, the true model orders were randomly chosen between 5 and 8. The cutoff order parameter passed to the method to be tested was 2 higher than the order of the true model. Various error measures relating to the coefficients, model order, conditional GC and pairwise GC were computed. These error measures were averaged over all samples of a given model size and time length. Performance was also tested with simulated noise added to the data. The results of the simulations are presented below.

Overall, the results showed that *SVARGS* gave highly accurate as well as truly sparse fits. SVARGS is considerably faster than glmnet-lasso at fitting the coefficients. Moreover, as it produces very few spurious coefficients (see Table 2), the CGC computation is speeded up by an order of magnitude or more by using SVARGS. As mentioned earlier, This makes SVARGS suited to compute CGC for much larger systems than existing semi-sparse methods such as glmnet-lasso.

For each of the model fits obtained above, the Conditional Granger Causality (CGC) was also computed. These were directly obtained from the VAR models using the procedures described in Supplementary Section 3.4. As mentioned in the introduction, one of the primary reasons for fitting VAR models to data is to then construct a functional connectivity network representation of the data using CGC (or another equivalent method). The network is constructed by taking the directed edge weight (from one variable/channel to another) to be the corresponding CGC value. In other words, the matrix of CGC values between different variables becomes the weighted adjacency matrix for the corresponding functional connectivity network. If a CGC value is zero, the corresponding directed edge is absent from the network. Ideally, we require a sparse set of CGC values (that is, a sparse CGC matrix) since that would correspond to a sparse functional connectivity network and our method implies that the CGC is sparse as a result of the sparsity of the VAR coefficient matrices. The Supplementary Fig. 4 shows the performance of the SVARGS method in computing CGC.

**Figure 1:**
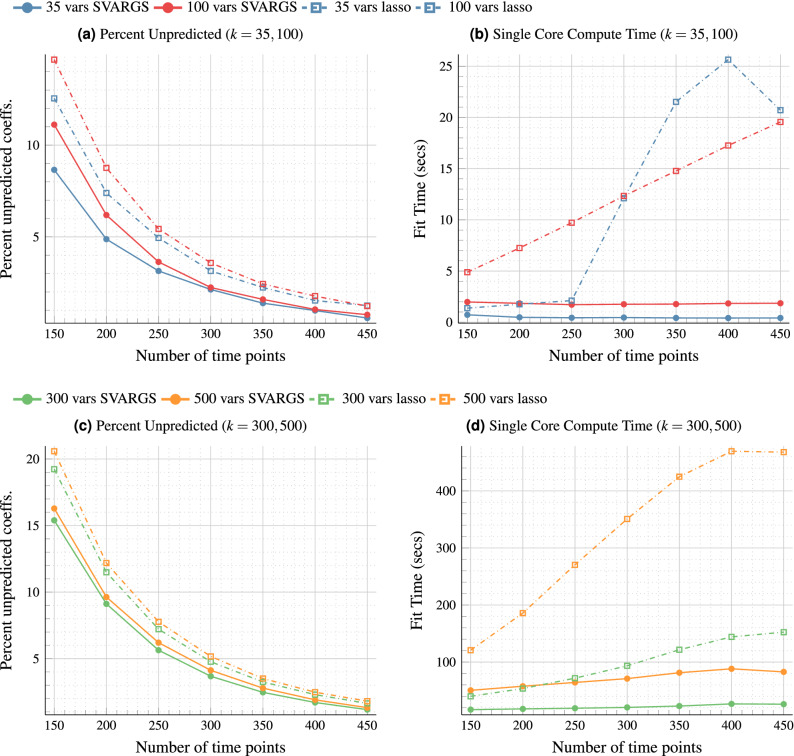
(**a,c**) (left column) Percent Unpredicted (false negative rate) = Number of true non-zero coefficients not in the fitted model)/(Number of true non-zero coefficients) for $k = 35, 100$ and $k = 300, 500$ respectively, averaged over 50 samples per data length. (**b,d**) (right column) Time taken in seconds (single processor core) to complete fit for $k = 35, 100$ and $k = 300, 500$ respectively, averaged over 50 samples per data length.

**Table 2:** Percent Spurious = Number of spurious predictions for every 100 true non-zero coefficients [For each system size, average over all simulations]

Method/num variables	35	100	300	500
SVARGS	12.8	7.0	5.6	5.4
Lasso	450.6	586.0	763.1	836.0

Figure 1 shows the performance of SVARGS for each model size and each time length. Figure 1a,c shows the percentage of unpredicted coefficients (percentage of false negatives) of both glmnet-lasso and our SVARGS method. Figure 1b,d show the computation speeds of both methods. Table 2 shows the percentage of spurious coefficients (percentage of false positives) for glmnet-lasso and SVARGS respectively for each of the four system sizes. The spurious percentages were more or less the same across the different data lengths so the average over data lengths is shown. Additional figures showing further measures of the fit quality can be found in Supplementary Section 4.

To test the performance of SVARGS when the data dimensionality more than an order of magnitude larger than the number of time points, we also generated a sparse stable VAR model with 2000 variables and 5 lags containing 7000 non-zero coefficients. The number of non-zero coefficients in the model at each lag were 1284, 1409, 1475, 1403 and 1429 respectively, and the average non-zero coefficient sizes at each lag were 0.21, 0.45, 0.61, 0.49 and 0.30 respectively. 250 time points were simulated using this model and SVARGS was used to fit this data with a cutoff order of 7. The accuracy measures of this fit are as follows: *True coefficient Count*: 7000; *Unpredicted (false negatives)*: 688; *Spurious*: 378; *Relative Weight of Spurious Coefficients*: 0.0776; *Normalized Rms Error in Predicted Coefficients*: 0.3307; *Mean Normalized Bias in Predicted Coefficients*: $-0.0857$; *Order Error*: 1.4953; *Correlation between True and Predicted Coefficients*: 0.9885. *Time required for fit*: $\approx 20$ min. The definitions of these error measures are provided in Supplementary Section 4.1

The performance results when noise is added to the data can be found in Supplementary Fig. 6. In addition to Conditional GC, we also computed pairwise GC values from the models obtained from our methods. Scatterplots showing the correspondence with pairwise GC as well as the consistency between these values and the summed pairwise spectra are shown in Supplementary Fig. 5.

## Application to experimental data and results

In this section, we apply our methods to experimental data. In our first application, we analyse multi-subject EEG data from the DEAP emotion database (section “Analysis of DEAP emotion dataset”). Network measures based on the resulting networks are used as features to classify subject assessed emotional states as high intensity or low intensity. In the second application, we use the same methods on fMRI data from the ADHD-200 dataset to distinguish and classify fMRI scans of ADHD and typically developing children and (section “Application to ADHD fMRI data”).

### Analysis of DEAP emotion dataset

We use SVARGS to detect emotional states from the DEAP emotion dataset^47^. In the DEAP experiment subjects were exposed to a set of 1-min long videos and asked to rank the levels of different emotions felt for each video. The emotion categories used were based on Russell’s Valence-Arousal scale^48^. The emotions of Valence (unpleasant to pleasant), arousal (lack of interest to excitement) and dominance (helplessness to empowerment) were ranked by each of 32 subjects on each of 40 videos on a scale of 1 to 9. In addition, the subjects also ranked how much they liked the video (liking) on the same scale as well as their level of familiarity with the video on a scale of 1 to 5. The gender of the subjects were split evenly between male and female, with ages between 19 and 37 with a mean age of 26.9. Details of where the videos were obtained, how the videos were selected and the criteria for extracting an emotionally charged 1 min part of the video, as well as a the experimental protocols used to conduct the experiment can be found in^49^.

For each of the 32 subjects, the experiment started with a 2 min baseline recording with the subject asked to relax while fixating on a displayed cross. Subsequently, the 40 videos were presented to the subject in two batches of 20. Each video was prefixed with a 5 s fixation before the start of the video and a 2 s postfix screen after the video informing the subject of their progress. After the postfix, the subjects recorded a self-assessment of their levels of valence, arousal, dominance, liking and familiarity of the video. After the first 20 videos, the subjects took a break and were offered refreshments. The second set of videos followed an identical protocol. The original DEAP EEG recording was performed at a sampling rate of 512 Hz with 32 Active AgCl electrodes placed according to the international 10–20 system. In addition to the the EEG electrodes, thirteen physiological signals were also recorded. In what follows, we used only the EEG signals as input to the SVARGS algorithm. After assessing the performance of SVARGS, features based on the peripheral signals were added to determine if they improved prediction accuracy.

#### DEAP data preprocessing

Although a preprocessed data set in the form of Matlab *.mat* files are made available via the DEAP website^47^, we nevertheless used the raw data provided and preprocessed it with the aid of three well known toolboxes. The first of these, the PREP pipeline^50^, is a “standardized early-stage EEG processing pipeline that focuses on the identification of bad channels and the calculation of a robust average reference”. This was followed by artifact removal using AMICA (Adaptive Mixture of Independent Component Analyzers, see^51^) and the ADJUST toolbox^52^ for artifact detection. Each of the two batches for each subject (consisting of about 25 min of EEG recording) were preprocessed separately—that is a total of 64 continuous recordings of about 25 min each were preprocessed. The preprocessing steps performed on each recording are outlined in the Supplementary Section 8.1:

#### DEAP features via SVARGS and CGC

Once the EEG recordings were preprocessed, each of the 64 recordings were separated into the the 20 individual videos that comprised the recording. This yielded a total of 1280 EEG trials (each trial corresponding to one video). Each trial had the 21 channels mentioned in Supplementary Section 8.1 and 15,360 time points. In order to ensure reasonable stationarity for running SVARGS, each trial was further split into 3 s epochs with an overlap of 1.5 s. This yielded 39 slightly overlapping epochs per video per subject.

The cutoff model order for running SVARGS was chosen, based on the results of sample runs of SVARGS with a large model order, as follows: For every video, 7 epochs were chosen randomly and the SVARGS algorithm was run with a cutoff order of 60 ($\approx 250$ ms) on each of these epochs. For each subject, this resulted in $40 \times 7$ (=280) sample models with a $(segment \times video \times lag)$ coefficient array for each model. Each of the 280 coefficient arrays *C* was summed as per the Eq. (6) below to produce a vector:

6 $$\begin{aligned} C_{as} = vecc\left( \sum _{i=1}^{7}\sum _{j=1}^{40}\left| C(i,j,.)\right| \right) \end{aligned}$$

where vecc() denotes columnization. $C^{as}$ was then averaged over the 280 such vectors for each subject to give a single vector $C^{as}_{mu}$ and the standard deviation $C^{as}_{std}$ was also computed. Finally the vectors $C^{as}_{mu}$, $C^{as}_{mu} + 3C^{as}_{std}$ and $C^{as}_{mu} - 3C^{as}_{std}$ were plotted and inspected for each subject, and a cutoff order beyond which $C^{as} + 3C^{as}_{std}$ was quite negligible (typically less than about $10^{-2}\max (C^{as})$) was determined visually for each subject. A common cutoff order that would work for all the subjects turned out to be about 25.

The SVARGS algorithm was then run on all of the epochs with a fixed model order of 25 which at 256 Hz corresponds to a history of about 100 ms. This resulted in 39 sparse VAR models per video trial for each one of the 39 segments. Following the authors in^49^, we used only, roughly, the second half of the video. This corresponded to SVARGS coefficients and computed CGC matrices for the last 24 segments.

As mentioned earlier, we want to use network measures as features in order to classify emotional states. We restrict ourselves to binary classifiers which distinguish between low and high intensities of the emotion dimensions described at the start of this section. For the dimensions of Valence, Arousal, Dominance and Liking, we categorized a level less than or equal to 5 as “low” and a level greater than 5 as “high”. For the Familiarity dimension, we set the boundary at 3.

For classification based only on the time domain features, we used the feature groups listed below. Binary network node-wise measures (1–6) in Table 1 were eliminated from consideration. In the following, $A_{q, s}$ denotes the (channels $\times$ channels) slice at lag *q* of the VAR(21) model for segment *s*.

**Time domain coefficient and CGC features**:

1. **CO**: VAR coefficients:
  Sum of squares over over lags ($A_{ss} = \sum _{s=16}^{39}{\sum _{q=1}^{25}{A^{2}_{q,s}}}$), ($21 \times 21$)
  Sum of positive coefficients over lags ($A^{+} = \sum _{s=16}^{39}{\sum _{q=1}^{25}{A^{+}_{q,s}}}$), ($21 \times 21$)
  Sum of negative coefficients over lags ($A^{-} = \sum _{s=16}^{39}{\sum _{q=1}^{25}{A^{-}_{q,s}}}$), ($21 \times 21$)
  Maximum coefficient over lags ($A_{min} = \sum _{s=16}^{39}{\max _{q=1}^{25}{A_{q,s}}}$), ($21 \times 21$)
  Minimum coefficient over lags ($A_{max} = \sum _{s=16}^{39}{\min _{q=1}^{25}{A_{q,s}}}$), ($21 \times 21$)
  Channel source and target coordinates of top 3 (x,y,z in normalized MNI) coefficients in $A^{+}$.
  Channel source and target coordinates of top 3 (x,y,z in normalized MNI) coefficients in $A^{-}$.
2. **NS-time**: Scalar network measures:
  Scalar measures (1–9 in Table 1) constructed from each $G_{s}^{0}$ and averaged over the segments *s*.
3. **NE-time**: Network edge measures:
  $G^{0} = \sum _{s=16}^{39}{G_{s}^{0}}$, ($21 \times 21$) (The CGC matrix)
  Channel source and target coordinates (x,y,z in normalized MNI) of top 3 CGC entries in $G^{0}$.
4. **NNw-time**: Weighted network node-wise measures:
  Nodal measures 1–14 in Table 1 constructed from each $G_{s}^{0}$ and averaged over the segments *s*.
  Nodal measure *Asymmetries*: Symmetric left-right channel differences of the nodal measures (9 differences).
  Nodal measure *Caudalities*: Symmetric front-back channel differences of the nodal measures (6 differences).

We also performed a separate classification based on spectral band strengths of the Conditional Granger Causality. A $21 \times 21 \times 128$ Conditional Granger Causality spectrum array for the frequency range 0 to 127 Hz was computed as described in Supplementary Section 3.4. The frequencies were partitioned into bands, $(0<= \delta< 4), (4<= \theta< 8), (8<= \alpha< 14), (14<= \beta < 30)$, $(30<= \gamma _{L} < 50)$ and $(50<= \gamma _{H} < 100)$. The CGC spectrum above 100 Hz was ignored. This yielded a further 24 CGC matrices $G_{s}^{band}$ for each one of the six EEG bands. Along with the full CGC matrices $G_{s}^{0}$, this gave 168 CGC matrices per segment. Each of these matrices were treated as a weighted directed functional connectivity network and various global and node-wise network measures (listed in Table 1) were computed for each of these CGC matrices and averaged over the segments. The feature groups **NS**, **NE** and **NNw** were computed now for each band. The list below specifies the additional feature groups used for the spectral band based classification . In the following, $A_{q,s}$ denotes the (channels $\times$ channels) slice at lag *q* of the VAR(21) model for segment *s*.

**CGC spectrum features**:

1. **NS-spect**: Scalar network band measures:
  Scalar measures 1–9 in Table 1 constructed from each $G^{band}$ $band \in \{\delta , \theta , \alpha , \beta , \gamma _{L}\}$ (1 feature per band).
2. **NE-spect**: Network edge band measures:
  $G^{band} = \sum _{s=16}^{39}{G_{s}^{band}}$ $band \in \{\delta , \theta , \alpha , \beta , \gamma _{L}\}$ (a $21 \times 21$ CGC band-spectral matrix).
  Channel source and target coordinates (x,y,z in normalized MNI) of top 3 CGC entries in each matrix $G^{band}$.
3. **NNw-spect**: Weighted network node-wise measures
  Nodal measures 1–14 in Table 1 constructed from each $G_{s}^{band}$ $band \in \{\delta , \theta , \alpha , \beta , \gamma _{L}\}$ (21 features per band).
  Nodal measure *Asymmetries*: Symmetric left-right channel differences of the nodal measures (9 per band).
  Nodal measure *Caudalities*: Symmetric front-back channel differences of the nodal measures (6 per band).

**Peripheral features (PERI)**: In addition to the above we computed features from the peripheral channels (EOG, EMG, GSR, Respiration, Plethysmograph and Temperature). The recording details for these are described in^47,49^. The only preprocessing that was performed for the peripheral features was removal of the electrical line noise. For the choice of peripheral features, we followed^49,53^. To compute the skin conductance slow response, and very slow response we used the methods outlined in^54^. To find eye blinks, we used the algorithm described in^55^. For the respiration pattern we used peak detection and spectral centroid. For heartbeats from the plethysmograph we used peak locations and the spectral peak. A total of 77 features were computed. These are listed below.

1. **Electro-oculogram (EOG) features**: *Horizontal EOG*: Mean, median, interquartile range, variance and total power; *Vertical EOG*: Mean, median, interquartile range, variance and total power, Average blink rate.
2. **Electromyogram (EMG) features**: *EMG Zygomaticus Major*: Mean, median, interquartile range, variance and total power. *EMG Trapezius*: Mean, median, interquartile range, variance and total power.
3. **Galvanic skin response (GSR) features**: *Skin Resistance*: Mean, median and interquartile rang; Mean of derivative (= Avg. slope); Mean of negative values of derivative (= Avg. decrease rate during decay time); Proportion of negative derivative values; Number of local minima; 10-band spectral energy in 0–2.4 Hz range. *Skin conductance*: Zero crossing rate and mean peak magnitude for skin conductance slow response (0.08–0.2 Hz); Zero crossing rate and mean peak magnitude for skin conductance very slow response (0–0.08 Hz).
4. **Respiration features**: Mean, std. deviation, median and interquartile range; Mean of derivative (= Avg slope); Breathing rate from avg breaths per minute; Breathing rate from spectrum using spectral centroid; Low (0.05–0.25) to high (0.25–5) band energy ratio; Maximum breath range; Mean peak to peak time in seconds; 10-band spectral energy in 0–2.4 Hz.
5. **Plethysmograph features**: (Measured via thumb): Average heartbeats per minute using beat peak locations; Heart rate variability (std. deviation of peak-to-peak intervals); Heart rate from spectral peak; Band energy in low (0.1–0.2 Hz) band; Band energy in high (0.3–0.4 Hz) band; Low (0.04–0.15 Hz) to high (0.15–0.5 Hz) band energy ratio.
6. **Skin Temperature features**: Mean, median and interquartile range; Mean of derivative (= Avg slope); Band energy in low (0–0.1 Hz) and high (0.1–0.2 Hz) bands.

#### DEAP feature preselection and classification

The measure selection was done by separating those measures that had high gain from those that have low gain (see section “Feature preselection”) and are described in Supplementary Section 7). The feature reductions thus obtained are described in Supplementary Section 8.2. The Supplementary Table 4 and Supplementary Table 3 show the final number of features used in each set of features tested.

#### DEAP classification performance with SVARGS and CGC

**Table 3:** Binary cross-validated classification accuracy on DEAP data set. The first row merely lists the proportions of the majority class for each emotion and sets the baseline accuracy. The second row shows, for each emotion, the cross-validated accuracy obtained by Koelstra et al.^49^. The third row shows the accuracies obtained using all the time-domain features obtained from our SVARGS method listed in section “DEAP features via SVARGS and CGC”. The fourth row is the same as the third except with the addition of the peripheral features. The fifth row shows the accuracy obtained with the SVARGS network scalar (NS-spect) and network edge band spectral features (NE-spect) and the last row shows the accuracy with NS-spect, NE-spect and the peripheral features. The feature group abbreviations are listed in section “DEAP features via SVARGS and CGC”. We used the the Adaboost-SAMME algorithm for classification, with 8-fold cross-validation aggregated over 8 runs. The accuracies shown for our methods is the average over 10 such meta runs of the modal accuracy in the last 75 out of 225 rounds of adaboost-SAMME. Any standard deviations are indicated in brackets.

Method	Valence	Arousal	Liking	Dominance	Familiarity
1. Baseline	56.56	58.91	66.95	62.11	56.64
2. koelstra et al.	57.60	62.00	55.40	NaN	NaN
3. SVARGS: time domain	74.76 (0.53)	66.47 (0.74)	70.92 (0.43)	70.41 (0.55)	69.70 (0.40)
4. SVARGS: time domain and PERI	75.13 (0.64)	66.55 (0.66)	69.75 (0.61)	70.60 (0.55)	69.88 (0.42)
5. SVARGS: NS-spect+NE-spect	75.15 (0.52)	67.46 (0.55)	70.40 (0.45)	72.01 (0.41)	69.94 (0.67)
6. SVARGS: NS-spect+NE-spect and PERI	75.71 (0.57)	67.82 (0.53)	70.47 (0.63)	71.60 (0.32)	69.73 (0.50)

The VAR and CGC features were then used as input to the Adaboost-SAMME classifier. The performance of the classifier using the time-domain features only as well as using CGC band-spectral is shown in Table 3. It is clear that our method gives much better classification results than the earlier results^49^. A more detailed table with accuracies using each of the the different feature groups is shown in Supplementary Table 2 and Supplementary Table 1.

From these tables it is clear that the different feature groups gave quite comparable accuracies when using our method. The SVARGS+CGC time domain features alone gave accuracies that are only slightly less than those obtained with the CGC spectral bands. Moreover, the addition of the peripheral features contributes only marginally to the accuracy. In addition, we found that including six personal characteristic features (*gender*, *age*, *handedness*, *head circumference*, *nasion to inion distance* and *left to right jaw distance*) made no significant difference to the accuracies obtained and hence the results for the classification with the personal characteristic features included are omitted here.

### Application to ADHD fMRI data

#### ADHD200 data

The ADHD-200 data (see^56^), consists of resting-state fMRI scans from 973 subjects across 8 independent imaging sites. For the 2011 ADHD competition, the data were divided into a training set consisting of 776 subjects and a holdout test set consisting of 197 subjects. The scans were obtained from children in the age group 7–21 years. Preprocessed versions of the data set (rois_1000) are available from nitrc.org^57^ and consist of the parcellated time series constructed by averaging the voxel time courses over each of 954 parcels (ROIS’s) in the grey matter areas of the MNI brain. Details of the preprocessing and parcellation strategy can be found at^58^. In addition phenotypic information such as gender, handedness, ADHD type, etc, is provided along with the datasets.

Of the 776 subjects in the training set, 108 subjects were dropped as all the fMRI scans from these subjects were marked as being of questionable quality. Only subjects who had at least one good scan were included. If there were multiple scans for a given subject, they were treated as a multi-trial time series and handled in the manner described in Supplementary Section 2.3.2. This reduced the size of the training set to 668.

Of the subjects having at least one good scan, 491 were typically developing children (TDC) and 285 were children and adolescents with ADHD. Table 4 below shows the number of subjects with usable scans in the training set:

**Table 4:** Number of subjects per site.

Site	BEIJING	KKI	NEURO.	NYU	OHSU	WASHU	PITTSBUR.	BROWN
TDC	116	58	22	91	40	66	33	0
ADHD	78	20	17	97	30	0	0	0
Total	194	78	39	188	70	66	33	0

#### Deconvolution of fMRI signals to correct for haemodynamic delay

Before we apply our method to determine functional connectivity using fMRI resting state signals, we address a question regarding the suitability of using the fMRI BOLD signal for this purpose. The BOLD (Blood Oxygen Level Dependent) technique first introduced by Seiji Ogawa^59^ measures certain changes to an externally applied magnetic field that are induced by variations in blood oxygen levels, volume and flow rates in the arteries of the brain. These BOLD signals are taken to indicate the level of electrical neuronal activity of the brain. However changes to the blood oxygen level take place relatively slowly, so the measured BOLD signal is some delayed and filtered version of the actual neuronal activity. This phenomenon is known as as Haemodynamic delay or distortion. Since Granger Causality and, in particular, Conditional Granger Causality depend on the timing of the signals, this places a question mark on the validity of using CGC as a proxy for functional connectivity in the brain.

For task-related fMRI, the issue of haemodynamic delay can be resolved by using explicit exogenous inputs to determine a *Haemodynamic Response Function (HRF)*^60,61^. For resting state fMRI we use a blind deconvolution method^62–64^. The main idea is to use larger than normal fluctuations in the BOLD signal as pseudo-events in order to construct a Haemodynamic response function (HRF). The original signal is then reconstructed by using the HRF to deconvolve the observed BOLD signal. The Matlab code for estimating the HRF and deconvoling the fMRI BOLD signal can be found in the Matlab rsHRF toolbox^65^.

We used the rsHRF toolbox for signal deconvolution prior to the analysis of fMRI time series data.

#### ADHD network measures via SVARGS and CGC

First, for each subject and each ROI channel, the resting state Haemodynamic Response Function was constructed and the channels deconvolved using the rsHRF toolbox mentioned at the beginning of section (4.2). Then the scan(s) for each subject were fit to a sparse VAR model of order 1 using SVARGS. Here the time resolution TR varied between 1.5 and 2.5 s for the different sites.

We note here that the SVARGS method can be used for the ADHD data with higher model cutoff orders. With the same ADHD data and a cutoff model order of 5, the predominant coefficients were at lag 1 and lag 2, somewhat smaller coefficients at lag 3, and much smaller coefficients at lags 4 and 5. Hence, technically speaking, a model order of 2 or 3 might have been more appropriate from the point of view of VAR modelling alone. However, the *classification accuracies* resulting from running the model fit and CGC network based classification pipeline are not significantly different for a cutoff order of 1 as compared to a cutoff order of 3. In addition, the authors in^14,66^ suggest that a model order of 1 is often sufficient for the analysis of fMRI data. Hence to save time and enable faster experimentation, a cutoff order of 1 was used.

For each subject, the resulting VAR model consisting of a $(954 \times 954)$ coefficient matrix and a corresponding $(954 \times 954)$ error covariance matrix was then used to obtain the Conditional Granger Causality matrix as described in Supplementary Section 3.4. Each of these 668 CGC matrices were treated as a weighted directed functional connectivity network and various global and node-wise network measures (listed in Table 1) were computed for each of these connectivity matrices. We examined whether Typically Developing Children (TDC) could be distinguished from ADHD children using these network measures.

#### ADHD feature set

For each subject network matrix, various scalar measures were computed. For each measure, this resulted in two feature for each subject. We also computed *node-wise* measures (each of the 954 ADHD200 rois treated as a node) for each of the 668 subjects. The scalar and node-wise network measures computed are shown in Table 1 (see^38^ for an explanation of these measures). We input these measures as features into a binary classifier. We considered the feature groups listed below. In the following, *C* denotes the coefficient matrix of the VAR(1) model. $C \odot C$ denotes the element-wise product matrix.

1. **CO** VAR coefficients:
  $C_{+}$: The positive VAR model coefficients—a ($954 \times 954$) matrix.
  $C_{-}$: The negative VAR model coefficients.
  $C \odot C$ (For a VAR(1) model, this is a special case of *Direct GC* (see^67^) ).
  $C \odot C$ based scalar, node-wise and edge-wise network measures.
2. **NS-time** Scalar network measures:
  CGC based Scalar network measures (1–9 in Table 1)
3. **NN-time** Weighted network node-wise measures:
  CGC based weighted node-wise measures (1–14 in Table 1)
4. **NE-time** Network edge measures:
  The conditional GC—a ($954 \times 954$) matrix.

#### ADHD feature preselection and classification

With 32 features at each node and 954 nodes, the number of node-wise features alone would be 30,528. While computationally quite feasible, it was nevertheless possible to improve the classification by reducing the number of nodes. Features corresponding to each node were treated as blocks, and selection of the blocks corresponding to each node was done by separating those nodes that had high gain from those that had low gain (see section “Feature preselection”). This resulted in the elimination of all but 24 roi nodes and subsequently only the node-wise network measures pertaining to these nodes were chosen as features. These high gain nodes could be regarded as the “most discriminatory” of the full set of 954 nodes. The number of node-wise features resulting from using these 24 nodes is then $24 \times 32 = 768$. Measure selection was similarly done by separating those measures that had high gain from those that have low gain. The Supplementary Section 7 describes the manner in which feature block separation is achieved.

For the several million edge-wise features, to reduce the large number of network edges and coefficients (**NE-time** and **CO**—many of them zeros), we only considered edges between the 24 nodes found above. Then in addition, for any such $24 \times 24$ matrix, only those entries (features) in each matrix were chosen that had had a non-zero value in at least *m* data entries (subjects) where *m* was the smallest number that was significant at a p-value of 0.01 under the hypothesis that the non-zero values were distributed randomly in the matrix. The value for *m* is easily computed since it would then follow a binomial (*N*, 0.1) distribution where *N* is the number of data instances.

These reduced set of features were then input as features to a classifier based on the Adaboost-SAMME algorithm. Table 5 shows the accuracies obtained with SVARGS+CGC in comparison with the best accuracies obtained in the ADHD global competition. Our method gives improved accuracies. The accuracies obtained in the ADHD global competition are shown for two cases: (i) using only fMRI features for classification; (ii) Personal Characteristic Data, PCD(1), consisting of the features *Site*, *Gender*, *Age*, *Handedness*, *Verbal IQ*, *Performance IQ* and *Full4 IQ*, for classification. We should note that for case (ii), using two additional personal characteristics, *IQ Measure* and *Full2 IQ*, gives a marginally better result on the training set (but not on the holdout set) than using PCD(1). But this is within the error bar of the result obtained using PCD(1) alone and is hence not included in the Table. In our method, we use both fMRI data and personal characteristic data PCD(1) for classification, an approach not used by any of the teams in the global competition.

**Table 5:** Binary (TDC/ADHD) classification accuracy of different methods on ADHD200 data set. The table shows cross-validated accuracies on the original training set and the accuracies obtained on the original holdout set. Standard deviations are shown in brackets. The first column shows the best accuracies obtained in the ADHD-200 Global Competition^68–70^. The second column shows the ADHD-200 Global Competition using PCD only. The best accuracies in the ADHD-200 Global Competition were obtained using only the personal characteristic data. The third and fourth columns show the accuracy using SVARGS+CGC features (4.2.4) and the adaboost-SAMME algorithm for classification with only fMRI and fMRI+PCD respectively. The last column merely lists the proportions of the majority class for each emotion and sets the baseline accuracy. The accuracies shown for our methods is the average over 10 meta runs. The accuracy on each each meta run is the aggregate over 8 runs of the modal accuracy in the last 75 out of 225 rounds of 12 fold cross-validation. The accuracy on the holdout set is the average over 20 runs at the 50th round of adaboost-SAMME.

	ADHD200 Comp. (fMRI only)	ADHD200 Comp. (PCD only)	SVARGS (FMRI only)	SVARGS (FMRI + PCD)	Baseline
ADHD Training Set	70.7 (6.2)	75.0 (4.5)	74.2 (1.2)	78.2 (0.37)	64.2
ADHD Holdout Set	60.5	69.0	65.8 (0.8)	73.4 (0.85)	55.0

## Discussion and conclusions

### Methods

We have presented a method for efficiently fitting sparse VAR models to high dimensional time series data. While in theory, our method works whenever the underlying model is sparse, it is particularly suitable for the case where the number of variables and/or the order is large while only a *moderate* number of time points is available for analysis and the density of the “important” coefficients of the underlying model is small. We must also make a note of the behaviour of the algorithm when the “true” model is dense. In this case, the algorithm may, nevertheless, yield a sparse model by picking only the most significant coefficients. The final model will then be sparse provided *N* is *sufficiently small*: Larger values of *N* will lead to denser models with correspondingly higher time complexity (see supporting information of Rangarajan et al.^36^ for an estimate of the time complexity of the algorithm). These qualifications are stated more precisely below.

If *N* is the number of time points, *k* the number of equations (variables) in the system and *p* the true order, the method requires that the following conditions hold:

1. The “true” number of non-zero coefficients (*m*) in any given equation is small compared to *N*. In practice, $m \le N/20$ seems to be quite sufficient. This implies that the total number of non-zero coefficients (*M*) in the system should satisfy the condition $M \le kN/20$. This is necessary for the validity of the arguments in Supplementary 2.3.8.
2. *N* must not be too small: Too few time points will affect the ability of the algorithm to detect coefficients. This is a result of the size of the confidence intervals for the coefficients discussed in Supplementary 2.3.8 and Supplementary 2.3.5. In practice $N \ge 100$ appears to be a reasonable condition.
3. For dense systems, in addition to conditions 1 and 2, it is appropriate to use SVARGS only if *N* is not too large: More precisely we must have $N \le {\mathscr {O}}(kp)$. In practice, this is not a major limitation since a long data recording can be (and often *should* be) analysed on a set of sliding time windows with a suitable length. Moreover the length of the time windows can be chosen, in some sense optimally, on the basis of the information criterion used in the SVARGS algorithm.

In Supplementary Section 3, we have outlined procedures that allow us to efficiently compute Granger causalities, as well as other quantities in the time and frequency domain, directly from the sparse VAR model without any further fitting. In particular, the results obtained in this manner for CGCs (and the resultant sparse functional connectivity network) are superior to thresholding CGCs using the theoretical asymptotic null distributions and the Geweke $\chi ^2$ test *after* obtaining a dense network from a dense VAR model.

Another significant advantage of our methods is the computation speed. On a desktop workstation, the SVARGS method is able to fit a sparse VAR model to 22,000-voxel data from the Human Connectome Project in less than 10 h. Moreover, the sparsity of our VAR models ensures the sparsity of the CGC matrix and the corresponding functional connectivity network (see Supplementary Section 5.2.1). This sparsity also enables faster and more numerically stable computation of the auto-covariance, particularly when using a fast algorithm like power doubling (see Supplementary Section 5.1). Computing the transfer function is also faster. As these are the major computational components of many auxiliary quantities (such as the power spectra and CGC spectra), our methods make the computation of these quantities for large systems feasible. Computing CGC values from the 22,000 variable sparse model for the data from the Human Connectome Project took less than 2 h. Further, the relatively low numbers of spurious coefficients means that the auxiliary quantities computed by us are also closer to their true values.

This ability to analyze very high-dimensional data within a reasonable time enables our methods to compute functional connectivity networks at both global and local levels. A demonstration of this ability was the analysis of ADHD data leading to network measures that are able to differentiate between ADHD and typically developing children. Yet another advantage of our methods is the following: If p-values are required, they can easily be computed theoretically along with the models, both for pairwise and conditional GC, as long as the original data length is known. This sidesteps the time-consuming computations required to create bootstrap distributions. For low dimensional simulated data ($< 5$ variables and a few lags) from random models, we have evidence that our method performs even better than exhaustive full regressor subset search based on the Akaike or Bayesian information criterion. This suggests that the statistical criterion we use to include or eliminate coefficients is more effective than *AIC* or *BIC*.

Finally, we would like to remark that our methods are not only applicable to neuroscience data (the major application area shown in this paper), but also to other time series data such as epidemiological data^36^, gene expression data, speech data, climatic data and financial data.

### Applications

Efforts to develop objective measures for the diagnosis of ADHD is an active ongoing area of research^71–74^. In addition to the VAR modelling and network based classification pipeline introduced in this paper, we hope that the application of our methods to ADHD may contribute, at least in a small way, to these efforts. However, developing objective diagnostic measures is not an easy task as the diagnosis is concomitant with, and contingent upon a specification of the neurological, behavioural and genetic markers that characterize ADHD, ie: the problem is partially circular. While evidence exists, based on the internal consistency of the diagnosis and follow-up of subjects, to show that ADHD is a valid psychiatric diagnosis^75,76^, many questions have been raised about the validity of ADHD diagnosis, particularly those based on behavioural and patient reported symptoms. Criticisms include subjectiveness (relying on evaluation of responses from patients), over-diagnosis (diagnosing the condition when it is not actually present)^77,78^, under-diagnosis (not diagnosing the condition when present)^79^ particularly in females. In addition, misdiagnosis of ADHD as some other condition and misdiagnosis of other conditions (such as Mood Disorders and Autism) as ADHD are also prevalent. The costs of the overdiagnosis and misdiagnosis^80^, for example in terms of the unknown long term impact of unnecessary medications, is also to be borne in mind.

We have no reason to assume that the the ADHD200 dataset is immune to the above criticisms. Hence we view the labels that have been assigned to the subjects in the dataset as approximations to the reality rather than the objective truth. In particular, we would caution here that the classifier may be training to the doctors diagnosis and not to any actual ADHD condition. So, based on the preceding concerns about the subjectivity of the diagnosis, it would be wise to treat very high classification accuracies with some skepticism. Nevertheless using our techniques on other ADHD datasets, even with moderate classification accuracies, would enable cross-validation of the features and ROI’s that are significant in the diagnosis of ADHD. As our classification pipeline is fully developed, this would not take much time and can form the subject of future work.

Regarding the EEG emotion classification on the DEAP dataset, firstly we note that here too, qualifications apply about the accuracy of the “true” labels. The labels for the DEAP dataset are based on Self Assessment Manikins or SAM’s^49^ page 5,^81^ and is a subjective measure based on the individuals own, possibly incomplete, dishonest or misunderstood assessment of their emotional state. Thus, as in the ADHD dataset, one should treat high classification accuracies based purely on EEG measurements with due skepticism. On the other hand, we have seen in this paper that in fMRI applications, the inclusion of personal characteristic data improved the classification accuracy. Thus, for potential real world applications^82^, it would not be unreasonable to expect significantly higher accuracies when EEG measurements are combined with other external signals and video data that captures facial expressions of the subjects^83,84^. Indeed, the DEAP recordings included facial recordings for some of the subjects, but they were not used in this work. In addition, features constructed from inputs to devices such as keyboards and mice, like timing and frequency parameters, can also play a role^85^. Thus the results of our application to the DEAP dataset should not be seen in isolation, but as a potential enhancement to features derived from a variety of other possible measurements.

The applications of EEG based emotion recognition include Extended Software Usability Testing (see for example^86^). This is particularly important for developing software for persons with motor disorders^87^. Another emerging area where EEG based emotion recognition is useful is Software Development Process Improvement^88^. In addition, E-healthcare^89^, E-education, Website Customization and Enhanced Video Game Development are all areas where EEG based methods, real-time or otherwise have been shown to be potentially useful^82^. An overview of emotion recognition applications based on EEG Brain Computer Interface systems can be found in^90^.

Finally, we remark that, in our applications, feature extraction on the full dataset occurred at a very coarse level or only to the extent of choosing biologically related groups of features (Supplementary Section 8.2 and sections “DEAP feature preselection and classification and “ADHD feature set”). This was to guard against tuning the set of extracted features too closely to the particular dataset. In the case of the ADHD dataset, the original holdout dataset was untouched until the feature extraction procedure was finalized. The results on the original holdout dataset (in Table 5) is evidence that excessive tuning of features did not take place. Experimenting with ignoring this constraint, both for the DEAP and ADHD datasets, expectedly led to far higher cross-validated accuracies than reported. A survey of EEG emotion recognition work based on the Russel Valence-Arousal[-Dominance] scale and a systematic overview of the types of features used by researchers and the corresponding accuracies obtained is presented in^91^.

## Supplementary Information

Supplementary Information.

## Data Availability

The data generated during this study are available from the corresponding author on reasonable request. Source code for the material presented as well as the url required to access updated versions of the code is included in the supplementary materials.
