# Supplementary material for: Sparse representations of high dimensional neural data
Source: Sci Rep. 2022 May 4;12:7295. doi: 10.1038/s41598-022-10459-7 (PMC9068763; doi:10.1038/s41598-022-10459-7)
Supplement: Supplementary file 1 — Supplementary Information. [file 41598_2022_10459_MOESM1_ESM.pdf]

Supplementary Information: Sparse Representations of High Dimensional Neural Data

Sandeep K Mody[1] and Govindan Rangarajan[1,2]

[1] *Deptartment of Mathematics, Indian Institute of Science, Bangalore, India*
[2] *Centre for Neuroscience, Indian Institute of Science, Bangalore, India*

February 2, 2022

Abstract

Section 1 provides supplementary information for the introductory section of the main manuscript. Section 2.1 provides a quick review of standard VAR modeling. Section 2.3 reviews the log likelihood ratio for comparing nested VAR models for testing the significance of Granger Causality, discusses the SVARGS algorithm in a manner applicable to blocks and has further comments on the algorithm such as choice of p_{entry} value. Section 3 describes all the quantities that can be computed directly from the VAR model without any further data analysis. Section 4 includes additional graphs showing performance measures for our methods. Section 5 outlines efficient methods for computing Autocovariance and the conditional GC matrix. Section 6 discusses the efficient implementation of the SVARGS.

1 Neuronal Networks

CoCoMac is a coordinate-independent collection of annotations compiled from "over 2,500 anatomical tracer injections" and from "over 400 published experimental studies" that captures 10,681 anatomical connectivity relations and 16,712 overlap (or mapping) relations between pairs of annotated brain regions. Based on these relations, Modha et al construct a network hierarchy consisting of 383 brain regions and $6,602$ directed edges spanning the entire Macaque brain. The undirected version of the network consists of $5,208$ edges which gives an average (undirected) degree of about 27.

2 VAR modeling

2.1 Standard method using Yule-Walker equations

We briefly summarize the standard method of estimating the VAR coefficients. The maximum likelihood method is used to derive estimates for the coefficients A_i. Suppose that the time series data $\hat{Y}$, composed of k variables, and N observations at regular time intervals, can be modeled by Main [2]. Denoting the observed values of ϵ_t by e_t, the Log-Likelihood, l_ϵ, of the observed noise (see [1, pg 346, section 5.4.1]), is given by:

$$l_\epsilon(e_{p+1}, \ldots, e_N, \Sigma_\epsilon) = \frac{(N-p)}{2} log \frac{|\Sigma_\epsilon^{-1}|}{(2\pi)^k} - \sum_{t=p+1}^{N} \frac{1}{2} \epsilon_t^T \Sigma_\epsilon^{-1} e_t \tag{2.1.1}$$

Maximizing (2.1.1) is clearly equivalent to minimizing the sum of squares, S_ϵ:

$$S_\epsilon(\{A_i\}) = \sum_{p+1}^{N} e_t^T \Sigma_\epsilon^{-1} e_t \tag{2.1.2}$$

The invertible linear relationship between the variables $\{\epsilon_{p+1}, \ldots, \epsilon_N\}^T$ and $\{Y_{p+1}, \ldots, Y_N\}^T$ implies that there is a one-to-one correspondence between the observed values $\hat{Y}$ of Y and the observed values e of ϵ, and

also that the Jacobian of the transformation from e to $\hat{Y}$ is constant (equal to 1 in this case). Hence choosing coefficients A_i that maximize the log likelihood of e is equivalent to maximizing the log-likelihood function of $\hat{Y}$. Therefore to minimize (2.1.2) with respect to the A_i, substitute from (2.1.1) to get:

$$S_Y(\{A_i\}) = \sum_{t=p+1}^{N} (Y_t - A_1 Y_{t-1} \ldots, -A_p Y_{t-p})^T \Sigma_\epsilon^{-1} (Y_t - A_1 Y_{t-1} \ldots, -A_p Y_{t-p}) \tag{2.1.3}$$

Minimizing (2.1.3) for A_i leads to the system of equations:

$$\begin{bmatrix} G_0 & G_1 & G_2 & \ldots G_p \\ G_1 & G_2 & G_3 & \ldots G_{p-1} \\ G_2 & G_3 & G_4 & \ldots G_{p-2} \\ \ldots & & & \\ \ldots & & & \\ G_{p-1} & G_{p-2} & G_{p-3} & \ldots G_0 \end{bmatrix} \begin{bmatrix} A_1 \\ A_2 \\ A_3 \\ . \\ . \\ A_p \end{bmatrix} = \begin{bmatrix} G_1 \\ G_2 \\ G_3 \\ . \\ . \\ G_p \end{bmatrix} \tag{2.1.4}$$

where:

$$G_i = \frac{1}{N} \sum_{t=p+1}^{N} Y_t Y_{t-i}^T \tag{2.1.5}$$

The $k \times k$ matrices $R(i)$ are the usual estimates for the auto-covariance function of the process Y_t. The equations (2.1.4) are the *Yule-Walker equations* using *estimated* autocovariances.

The solution obtained by (2.1.4) above is often not satisfactory, since for large systems with only moderate amounts of data, the amount of noise in the coefficients makes identification of the actual non-zero coefficients difficult. Moreover, variations on ths method based on identification of the best model order based on information criteria such as Akaike Information Criterion (AIC) or Schwartz Bayes Criterion (SBC) all lead to the same issue of noise in the coefficients.

For the procedures to be described, we need to use regression with the lagged values of $\hat{Y}$ as predictors. Below, we show this kind of regression is valid and is a generalization of the Yule-Walker equations.

2.2 Coefficients with constraints via regression

The coefficients A_i obtained from (2.1.4) are also exactly the coefficients that are obtained if we used linear Least-Squares regression on the data with the lagged values of $\hat{Y}$ as predictors. More precisely, if $y^{(r)}, r = 1, 2, \ldots, k$, are the individual components of Y, set:

$$x_i^{(r)} = y_{t-i}^{(r)} \tag{2.2.1}$$

$$X = \bigcup_{r=1}^{k} \{x_1^{(r)}, x_2^{(r)}, \ldots, x_p^{(r)}\} \tag{2.2.2}$$

If for each $r = 1, 2, \ldots, , k$, we perform a linear Least-Squares regression of the observed values $\hat{y}^{(r)}$, of $y^{(r)}$, on the observed values $\hat{X}$ of X, the coefficients obtained are identical to those from (2.1.4). This fact is easy to see from the standard formula for the linear regression coefficients:

$$X'X\beta_r = X'y^{(r)}$$

where $\beta_r = [a_1^r, a_2^r, \ldots, a_p^r]^T$ is the column vector of coefficients obtained by concatenating the rth rows of the matrix coefficients $A_1, A_2, \ldots, A_p$. Putting the k equations above together, we get:

$$X'X[\beta_1, \beta_2, \ldots, \beta_k] = X'Y \tag{2.2.3}$$

which on inspection will be seen to be identical to (2.1.4).

Now suppose that, there are linear constraints on the coefficients $\{A_q\}$, of the form:

$$C[a_{qrs}] = c \tag{2.2.4}$$

Here a_{qrs} is the element from the rth row and s^{th} column of A_q, C is a matrix with lk^2p columns, and c is a column vector of length $l < k^2p$ where l is the number of constraints. The column vector $[a_{qrs}]$, of length k^2p, is formed by running over all q, r, s.

This represents a general linear relation between the set of all coefficients. For such constrained coefficients, we can use the *Lagrange Multiplier Method* to minimize (2.1.3) taken along with (2.2.4).

The question arises whether we can continue to estimate the coefficients A_i as before, using regression on the equations for the individual components $y^{(r)}$. The answer is **no** in general but **yes** if the constraints are of a special form. In general coefficient estimates obtained by using the lagrange multiplier method on equations (2.1.3) and (2.2.4) are **not** the same as those obtained by performing linear Least-Squares regression on the individual equations. This is because Least-Squares regression on individual components will fail to account for inter equation constraints. However in the special case when (2.2.4) consists only of **intra**-equation constraints of the form:

$$C_r[\hat{a_1^r}, \hat{a_2^r}, \ldots, \hat{a_p^r}]^T = c_r, r = 1, 2, \ldots, k \tag{2.2.5}$$

where each C_r is a matrix with kp columns and $\tilde{l_r} < kp$ rows, a_q^r is the rth row of the matrix A_q and each c_s is a vector of length $\tilde{l_s}$, the lagrange multiplier equations simplify to give the individual regression equations as before. In particular, subset constraints of the form:

$$a_{qrs} = 0 \tag{2.2.6}$$

for particular sets of indices $\{q, r, s\}$, are trivially of the form (2.2.5). Solving for the coefficients A_i is equivalent to performing a separate linear regression on each of the individual equations. For the rth equation, only those regressors in the set X (see (2.2.1) that have non-zero coefficients need to be considered.

2.3 SVARGS

2.3.1 Maximum Likelihood for VAR(p) model

We denote the Maximum Likelihood Estimate of A_i by $\hat{A}_i$. The value of the maximum likelihood of the observed data can now be found by substituting these estimates into equation (2.1.1). The value of the log likelihood evaluated at $\hat{A}_i$ is:

$$l(\Sigma_\epsilon, \{\hat{A}_i\}) = \frac{(N-p)}{2} log\left[\frac{|\Sigma_\epsilon^{-1}|}{2^k\pi^k}\right] - \sum_{t=p+1}^{N} \frac{1}{2} e_t^T \Sigma_\epsilon^{-1} e_t \tag{2.3.1}$$

where $e_t = y_t - \sum_{i=1}^{p} A_i y_{t-i}$

Now in practice, we do not know the value of the theoretical error covariance Σ_ϵ in advance. So we have to treat it as a parameter to be estimated. We calculate its maximum likelihood estimate from the expression for the log-likelihood. Equations (2.1.3) already maximize the likelihood with respect to $\{A_i\}$ and moreover the estimate for $\{A_i\}$ is independent of Σ_ϵ. We therefore only need maximize (2.3.1) with respect to Σ_ϵ. We do this by differentiating (2.3.1) with respect to Σ_ϵ^{-1} and equating to 0. To do this we calculate the derivative with respect to the ij^{th} component σ_{ij} of Σ_ϵ^{-1} and use the symmetry of Σ_ϵ. We use the fact that for any matrix U, the differential of the determinant, $|U|$ with respect to its ij^{th} entry u_{ij} is $(-1)^{i+j} M_{ij} dl_{ij}$ where M_{ij} is the minor of u_{ij}. Also, the differential of $e_t^T U e_t$ with respect to u_{ij} is just $(e_t^T e_t)_{ij} du_{ij}$. We then get:

$$\frac{\partial l(\Sigma_\epsilon, \{A_i\})}{\partial \Sigma_\epsilon^{-1}} = \frac{(N-p)}{2}\Sigma_\epsilon - \sum_{t=p+1}^{N} \frac{1}{2} e_t e_t^T = 0$$

and hence the estimate for Σ_ϵ which maximizes the likelihood is:

$$\Sigma_\epsilon = \frac{1}{N-p}\sum_{t=p+1}^{N} e_t e_t^T \qquad [\quad = \Sigma_e \quad] \tag{2.3.2}$$

Substituting this in (2.3.1), and after a few simplifications, we get for the maximum likelihood:

$$l(\{\hat{A}_i\}) = \frac{(N-p)}{2} log\left[\frac{|\Sigma_e^{-1}|}{2^k\pi^k e^k}\right] \tag{2.3.3}$$

$$= -\frac{(N-p)}{2}[log(|\Sigma_e|) - k(1+log(2\pi))] \tag{2.3.4}$$

2.3.2 Time Series with multiple trials

To fit multitrial data with r trials, interleave the trials by placing, for every channel i and time point j, all the trial values at (i,j) adjacent to each other in the same row. and use regressors that are lagged by multiples of r only. For each equation, this procedure can easily seen to be theoretically equivalent to fitting the coefficients by inverting the average, over all realizations, of the regressor covariance covariance matrix.

2.3.3 Likelihood Ratio Test and Wilk's Theorem

Suppose we have two models M_0 and M_1 for a data set given by $\{y_1, y_2, \ldots, y_N\}$. Let the models correspond to parameter sets $\mathsf{A}^{(0)}$ and $\mathsf{A}^{(1)}$ respectively. The *Likelihood Ratio*:

$$\Lambda(\mathsf{A}^{(1)} : \mathsf{A}^{(0)}) = \frac{L(\Sigma_\epsilon, \mathsf{A}^{(1)})}{L(\Sigma_\epsilon, \mathsf{A}^{(0)})} \tag{2.3.5}$$

is a measure of the superiority of the model M_1 over the model M_0. If $\Lambda(\mathsf{A}^{(1)} : \mathsf{A}^{(0)}) > 1$, we can consider model M_1 to be better than model M_0. Hence we need to determine if the statistic $\Lambda(\mathsf{A}^{(1)} : \mathsf{A}^{(0)})$ is *significantly* larger than unity. For this we need its probability distribution which, in general, can be difficult or complicated to find. However, in the case when $\Lambda(\mathsf{A}^{(1)} : \mathsf{A}^{(0)})$ is a ratio of maximum likelihoods, the following result known as Wilk's theorem ([2]), enables us to approximate its distribution.

Theorem 1 (Wilk's Theorem)**.**
Let $Y = \{Y_1, Y_2, \ldots, Y_N\}$ be a random sample having joint probability distribution $f(y, \boldsymbol{\theta})$, where $\boldsymbol{\theta} = \{\theta_1, \theta_2, \ldots, \theta_m\}$ are unknown parameters. let $\hat{\boldsymbol{\theta}}$ be the Maximum Likelihood Estimate (MLE) of $\boldsymbol{\theta}$ within some open set $\boldsymbol{\Omega}$ in $\mathbb{R}^m$. Let $\hat{\boldsymbol{\theta}}_\mathbf{0}$ be the MLE of $\boldsymbol{\theta}$ in $\boldsymbol{\Omega_0} \subset \boldsymbol{\Omega}$, where $\boldsymbol{\Omega_0}$ is open in $\mathbb{R}^{m-\lambda}$, $\lambda > 0$. Assume that the maximum likelihood estimates of $\boldsymbol{\theta}$ have asymptotically ($N \to \infty$) normal distributions. Let $\mathsf{H}_0 : \boldsymbol{\theta} = \boldsymbol{\theta_0}$ be the null hypothesis. Then under H_0, the log likelihood ratio:

$$r_\lambda = 2log(\Lambda(\boldsymbol{\theta} : \boldsymbol{\theta_0}))$$

is asymptotically, distributed according to the $\chi^2(\lambda)$ distribution, i.e.:

$$\lim_{N\to\infty} P(r_\lambda < r) = F_{\chi^2(\lambda)}(r)$$

where $F_{\chi^2(\lambda)}$ is the CDF of the $\chi^2(\lambda)$ distribution.

2.3.4 Determine significance of estimated GC value

The concept of Granger Causality can be extended to use it in a lagwise manner instead of just a variablewise manner. Given the time series Y as above, suppose we have some existing model M_0 for it. Given the existing coefficients of the model, consider the Likelihood ratio restricted to a block of components $Y^{to} = \{Y^{(j_1)}, Y^{(j_2)}, \ldots, Y^{(j_s)}\}$ after entry of another disjoint block of components $Y^{from} = \{Y^{(i_1)}, Y^{(i_2)}, \ldots, Y^{(i_r)}\}$ at some lag q. From (2.3.3), this is given by:

$$R = (N-p_0)log(|\Sigma_{to}|) - (N-p)log(|\tilde{\Sigma}_{to}|) + 2k(p-p_0)(1+log(2\pi)) \tag{2.3.6}$$

where $|\Sigma_{to}|$ is the error covariance of the *to* block in the original model M_0, and $|\tilde{\Sigma}_{to}|$ is the error covariance of the *to* block after adding the coefficient locations of the $from$ block at lag q and refitting the model. p_0 and p are the orders of the model (restricted to the *to* block) before and after adding the new coefficients. The third term in equation (2.3.6) is a penalty term somewhat analogous to the penalty term found in information criteria such as AIC. In our case, we fix a maximum possible lag, p_{max} beforehand and use only data starting from $p_{max}+1$. This also has the advantage that genuine initial data is available from the data upto lag p. (2.3.6) then reduces to:

$$R = (N - p_{max})(log(|\Sigma_{to}|) - log(|\tilde{\Sigma}_{to}|) \tag{2.3.7}$$

$$= (N - p_{max})gc_{from \to to} \tag{2.3.8}$$

Because the coefficients are found using linear Least-Squares regression, (2.3.7) is a ratio of maximum likelihoods, and hence it follows from Wilk's theorem above that $(N - p_{max})gc_{from \to to}$ has asymptotically a $\chi^2(\lambda)$ distibution, where λ is number of components in the $from$ block. We can therefore test the GC statistic in exactly the same way as the Likelihood ratio. If the blocks are atomic (that is have only one variable), then this Granger Causality statistic is closely related to the F-statistic in linear regression. The F-statistic has an $F(1, n - \lambda_0 - 1)$ distribution, where λ_0 is the number of coefficients in the model M_0. For large n, this distribution approximates the $\chi^2(1)$ distribution. The results in section Main [3] show that GC statistic above is an effective way to determine all the significant coefficients at different lags.

2.3.5 Confidence intervals for coefficients

The finite number of time point implies some uncertainity in the size of the coefficients obtained. In addition, this uncertainity, places a threshold of the size of the smallest coefficients that can be detected by the algorithm. In order to find the uncertainity in the computed coefficients, first note that for a random variable Z that is the sum of squares of identically normally distributed random variables χ_i

$$\zeta = \frac{1}{N}\sum_i \chi_i^2 \tag{2.3.9}$$

the standard deviation s_ζ of ζ is $\frac{1}{\sqrt{N}}s_\chi$, where s_χ is the common standard deviation of the variables χ_i. Moreover Z is asymptotically normally distributed as $N \to \infty$. In our case, where N is at least 100 or more, we can take Z to be approximately normal.

We show in section Supp[6], equation Supp[(**??**)] that when a new coefficient c is added at some stage of the fit, the following equation holds:

$$\Delta\sigma = c^2 \bar{x}^T (I - H_0)\bar{x}$$

Here $\bar{x}$ is the new regressor associated with the location of the coeffient c, $\Delta\sigma$ is the change in the sum of squares of residuals due to the addition of the new coefficient and H_0 is given by:

$$H_0 = Q_0 Q_0^T$$

where Q_0 is the orthonormal matrix in the QR factorization of the the set of regressors X_0 before the new regressor was added. Now we have:

$$\frac{1}{N}\Delta\sigma = \frac{1}{N}\sum_i (e_i^2 - e_{0i}^2)$$

where e_{0i} and e_i are the components at time i of the residuals $\bar{e}$ and $\bar{e}_0$, respectively, before and after the entry of c. So we can write:

$$c^2 = \frac{\sum_i e_i^2}{\bar{x}^T(I - H_0)\bar{x}} - \frac{\sum_i e_{0i}^2}{\bar{x}^T(I - H_0)\bar{x}} \tag{2.3.10}$$

Now note that the response variable $\bar{y}$ and the lagged regressors X are jointly normally distributed. Hence the residuals $\bar{e}$ in the first term, which are a linear combination of the response variable $\bar{y}$, the regressors

in X and the newly added regressor $\bar{x}$ are jointly normally distributed. In addition the residual vector $\bar{e}$ is orthogonal to $\bar{x}$ which implies that $\bar{x}$ and and $\bar{e}$ are independent random variables. Now it can be shown (see for example [3, Ch. 9, section 4]), that if M is an idempotent matrix, then $\bar{x}^T M \bar{x}$ has a $\chi^2(N-r)$ distribution where r is the rank $(= tr(M))$ of M. Since $I - H_0$ is idempotent, it follows that the denominator $\bar{x}^T(I-H_0)\bar{x}$ of (2.3.10) has a $\chi^2(N-r)$ distribution. (Note incidentally that, by our assumption of sparsity, r is small compared to N). In addition the numerator of the first term has a $\chi^2(N)$ distribution. In view of the independence of $\bar{e}$ and $\bar{x}$, the numerator and denominator are also independent (H_0 is also formed from lagged regressors) and hence the first term in (2.3.10) has an $\mathcal{F}(N, N-r)$ distribution with variance $\mathcal{O}(\frac{1}{N^2})$.

For the second term of (2.3.10), we don't have independence of the numerator and denominator and hence we simply treat them as a ratio of two χ^2 distributions. The variance of the numerator and denominator are $\frac{2\sigma_e}{N}$ and $\frac{2\sigma_x}{N-r} \approx \frac{2\sigma_x}{N}$. Taking the square root of (2.3.10) and making the obvious approximations, it follows that the uncertainity in the estimation of c is given by:

$$\sigma_c \approx 2\sqrt{\frac{2}{N}}\left(\frac{s_e}{s_x}\right) + \mathcal{O}(\frac{1}{N}) \tag{2.3.11}$$

$$\approx 2\sqrt{\frac{2}{N}}\left(\frac{s_e}{s_x}\right) \tag{2.3.12}$$

where s_e and s_x are the common standard deviations, respectively of the e_i and x_i.

2.3.6 Entry p-Value for SVARGS

We provide a rationale for our choice of p-values for coefficient entry in the SVARGS algorithm. The argument is heuristic and we relate it to the concept of the False Discovery Ratio (FDR). First note that for each coefficient entry, we test k^2q coefficients (over all equations). For any particular coefficient whose true value is 0, the probability of a false entry at that step is p_{entry}. Now, if a coefficient was falsely entered, but the true value of its likelihood statistic is unity, then the probability that it will remain at the removal step is p_{exit}. For any such coefficient (whose true value is 0), let $\tilde{p}_n$ be the probability that it is in the entered state at the nth step. Then we have the following recursion:

$$\tilde{p}_n = p_{exit}\tilde{p}_{n-1} + p_{entry}(1-\tilde{p}_n) \tag{2.3.13}$$

This can be rewritten as:

$$\tilde{p}_n = a\tilde{p}_n + b$$

where $a = p_{exit} - p_{entry}$ and $b = p_{entry}$

Solving the recursive equation upto the nth step gives:

$$\tilde{p}_n = \frac{1-a^{n-1}}{1-a}b$$

Since a is small, this becomes:

$$\tilde{p}_n \approx p_{entry} \tag{2.3.14}$$

which is what one might naively assume without going through the above calculation. In other words, our naive intuition that p_{entry} is the probability of a zero coefficient being falsely detected is justified. The expected number of false entries $E_{\tilde{n}}$ is therefore:

$$E_{\tilde{n}} = k^2q \times p_{entry}$$

We would like $E_{\tilde{n}}$ to be small compared to the final number of coefficients. How small is decided by the choice of the False Discovery Rate, FDR. In terms FDR, the number of false detections is $FDR \times M$. Equating this to $E_{\tilde{n}}$ above, we get:

$$k^2q \times p_{entry} = FDR \times M$$

and hence:

$$p_{entry} = \frac{FDR \times M}{k^2 q} \tag{2.3.15}$$

We may choose FDR based on the expected number of non-zero coefficients in the final model (For example if we know the approximate sparsity beforehand, one might want to take $FDR = \mathcal{O}(1)/M$).

The approach we take, after choosing the FDR, is to think of the corresponding value in 2.3.15 as just indicative of the best p_{entry} value. In our algorithm, we choose a range of values around this value. At the start of the algorithm we use the lowest value in the range and then progressively increase it (as coefficient addition gets exhausted) upto the maximum value in the range (we can also think of this as testing a range of FDR values instead of a range of p_{entry} values). The best value is then chosen using the extended SBC information criterion. The additional computational cost of this approach is very moderate because we are not refitting the model for every value of p_{entry}.

A somewhat more time-intensive alternative is to compute the model for a suitable range of values of p_{entry} and then use some form of cross-validation to determine the best value.

2.3.7 Extended SBC criterion

In our experiments with different information criteria for model selection for the SVARGS algorithm, we found that both the AIC as well as the SBC criteria were too liberal in their inclusion of coefficients. The huge number of spurious coefficients also resulted in an unacceptable slowing down of the algorithm. The *Extended SBC* criterion ([4]) is based on somewhat different assumptions about the model. The usual SBC criterion is derived on the basis of the prior probability of each coefficient subset being equal. For large models, this implies that larger models are assigned a higher prior probability (since the number of different subsets for a given number of coefficients increases exponentially with the number of coefficients). In [4], the extended SBC criterion is derived based on assigning equal prior probabilities to different model sizes rather than different coefficient subsets. The resulting criterion is:

$$SBC_X(m) = SBC(m) + 2\gamma log(\tau(M,m)), \quad (0 \leq \gamma \leq 1) \tag{2.3.16}$$

Here SBC is the usual term for Schwartz-Bayes information criterion ($= -2(LogLikelihood) + mlog(N)$). The second term is the adjustment for the different assumed prior. m is the complexity of the model and the quantity $\tau(M,m)$ is the number of different subsets of coefficients of size m that can be constructed from a pool of coefficients of size M.

γ is a number between 0 and 1 and must be chosen appropriately depending on whether $\kappa = log(M)/log(N)$ is approximately 1 or more ($\gamma = 1$), or κ is somewhat less than 1 ($\gamma = 0.5$) or κ is well below 1 ($\gamma = 0$). We used a smooth approximation to the step function passing through these points.

The criterion is is shown to be consistent and satisfying the condition that the probability of selection of a model other than the true model approches zero as the number of data points approaches ∞.

2.3.8 SVARGS coefficient confidence and size threshold

The finite number of time points implies some uncertainity in the size of the coefficients obtained. In addition, this uncertainity places a threshold of the size of the smallest coefficients that can be detected by the algorithm. In 2.3.5 we determine the uncertainity, σ_c, in the estimated size of the coefficients. Coefficients not much larger than σ_c have a fair chance of not being detected or being eliminated due to the natural variance of the finite sample of size N. Coefficients whose size is more than twice σ_c are almost certain to be detected. Based on σ_c, a rule of thumb for the threshold on the size of detectable coefficients can be taken to be:

$$z_c = 4\sqrt{\frac{2}{N}}\left(\frac{s_e}{s_x}\right) \tag{2.3.17}$$

where s_e is the standard deviation of the residual variance of the equation containing the coefficient c, and s_x is the standard deviation of the variance of the regressor corresponding to c. In general, the greater the explanatory power of the regressors, the smaller the variance of the residual sum of squares and the better is the detectability of the coefficients.

2.3.9 General comments on SVARGS

Note that the SVARGS fit need not necessarily give the best subset of predictors in any well-defined sense. This is a well-known drawback of stepwise regression in general. There may be approximate redundancies among the lagged predictors, so that a best subset of predictors may not even exist in a strictly mathematical sense. At best, one can say that it gives coefficient locations at which the (penalized) likelihood has a local maximum. Also note that the p-values obtained as the fit progresses are only used for model comparison in order to determine the next coefficient location to add or remove. These p-values are entirely dependent on the path taken by the fitting process and only the *relative* size of consecutive p-values are relevant for the progress of the fit. Thus these p-values are not used in any way to rank the final set of model coefficients. For the intended applications of this method, this is perfectly alright since we are mainly interested in the question: "What is the (relatively) small set of significant interactions, delayed or otherwise, between variables". A rough idea of the importance of the interactions can be gleaned from the (conditional) Granger Causality strengths. Moreover if, for some reason, it is necessary to rank the coefficients, one can compute p-values from the confidence intervals (computed in section 2.3.5) for the final set of coefficients .

In view of the above, we also observe that the final model obtained was fairly robust with respect to a range of FDR values. A higher FDR led to more spurious coefficients but the prediction rate did not vary much as long as the FDR was not too large. This was found to be true, both, for data generated from sparse models as well as for real data. In our opinion, this indicates that in our applications (and we suspect often in many other real world applications), there is a wide gulf between those interations that are significant and those that are not, with very little in the grey area. Note however that the FDR increases the computation *time* by increasing the coefficient density as well as the *reversal-ratio* - the ratio of the number of backward steps to the total number of non-zero coefficients (see supporting information of [5] under the section *time complexity*). In particular, having a somewhat low FDR can speed up the fit considerably.

In addition, the method works best, both in terms of quality of fit and time complexity, when the final model is known to be sparse with the number of non-zero coefficients being of order $\mathcal{O}(mk)$ with $m \ll N$ and $m \ll kq$ (where k is the number of variables in the model and q is the number of lags and N is the number of time points). It is *not* suitable for dense or even semi-sparse models. As the final coefficient count increases, the time complexity of the method degrades more than proportionally with respect to the final number of coefficients. See the supporting information in [5] under the section on *time complexity* for a discussion of the time complexity of the algorithm.

3 Quantities derived from VAR model

In order to find the Granger causalities (in both the time and frequency domains) from the fitted model, we first need to compute the following quantities using the fitted model:

1. ACVF: Matrix of variance and cross-covariance at any given lag.
2. Sub-Model Coefficients: Given a subset of variables and a subset of lags, compute a VAR model as if all the "data" is to be explained by only the specified subset of the original set variables and the specified subset of the original set of lags.
3. Transfer Function: Transfer function of the operator associated with the VAR model.
4. Spectrum: Matrix of power and cross spectra at any given frequency).

To compute the Granger causality strengths, we ony require (1) and (2). For the GC spectrum we require (2) as well as (3) and (4). Under a certain technical condition that we shall state at the end of this section

(section 3.5), the integral over all frequencies of the GC spectrum is identical to the GC strengths. In the following sections, we describe how to calculate these quantities and subsequently the granger causalities.

3.1 ACVF, Transfer Function and Spectrum

Given the VAR model Main [2] of order p in k variables, one can construct an equivalent VAR model of order 1 in kp variables using the transformations:

$$x_t^{(j+1)} = y_{t-j}, \quad j = 0, 1, \dots, (p-1) \tag{3.1.1}$$

This yields the extended model:

$$\mathsf{X}_t = \mathsf{A}\mathsf{X}_{\mathsf{t-1}} \tag{3.1.2}$$

where the extended vector X is the column vector of length kp and the extended matrix A is a $(kp \times kp)$ coefficient matrix:

$$\mathsf{X} = \begin{bmatrix} x^{(1)} \\ x^{(2)} \\ . \\ . \\ x^{(p)} \end{bmatrix}, \qquad \mathsf{A} = \begin{bmatrix} A_1 & A_2 & & \dots & A_p \\ & \ddots & & & 0 \\ & & I & & \vdots \\ & & & \ddots & 0 \end{bmatrix}$$

As such, the form 3.1.2 is not efficient for direct numerical computation. However it can be used to conveniently derive more efficient formulas to compute the above mentioned quantities. We do this below.

3.1.1 Autocovariance Function (ACVF)

From the extended order 1 model above, the equations for the autocovariance G can be found by multiplying both sides of the equation first by $\mathsf{X_t}$ and then by $\mathsf{X_{t-1}}$ and taking expectations on both sides. The resulting two equations are:

$$\mathsf{G} = \mathsf{A}\mathsf{G}'^{\mathsf{T}} + \Sigma$$
$$\mathsf{G}' = \mathsf{A}\mathsf{G}^{\mathsf{T}}$$

which can also be written as a single equation:

$$\mathsf{G} = \mathsf{A}^{\mathsf{T}}\mathsf{G}\mathsf{A} + \Sigma \tag{3.1.3}$$

The matrix Σ is zero except for the top-left $(k \times k)$ block which is the same as the noise covariance of the original model in Y:

$$\Sigma = \begin{bmatrix} \Sigma_\epsilon & 0 \\ 0 & 0 \end{bmatrix}$$

and G and G' are block-toeplitz matrices representing the extended autocovariances at lag 0 and lag 1 respectively:

$$\mathsf{G} = \begin{bmatrix} G_0 & G_1 & \dots & G_{p-1} \\ G_{-1} & G_0 & \dots & G_{p-2} \\ \dots & & & \\ \dots & & & \\ G_{-p+1} & G_{-p+2} & \dots & G_0 \end{bmatrix}, \qquad \mathsf{G}' = \begin{bmatrix} G_{-1} & G_0 & \dots & G_{p-2} \\ G_{-2} & G_{-1} & \dots & G_{p-3} \\ \dots & & & \\ \dots & & & \\ G_{-p} & G_{-p+1} & \dots & G_{-1} \end{bmatrix}$$

Here G_j represents the autocovariance at lag j of the original variable Y_t. Note that $G_{-m} = G_m^T$. Substituting these into the equations for the extended autocovariance and performing the block multiplications, we get, after some matrix algebra, the following p equations:

$$G_j = \sum_{i=1}^{j} A_i G_{j-i} + \sum_{i=j+1}^{p} A_i G_{i-j}^T, \qquad j = 1, 2, \ldots, p-1 \tag{3.1.4}$$

$$G_0 = \sum_{i=1}^{p-1} A_i G_i^T + A_p \sum_{i=1}^{p} \{A_i G_{p-i}\}^T + \Sigma_\epsilon \tag{3.1.5}$$

For a description of the methods used to solve (3.1.3) or (3.1.4) see section 5.

Note: In the remainder of this section, we set $\alpha(\omega) = e^{2\pi i\omega}$.

3.1.2 Transfer Function

The Transfer function, $H_p(\omega)$ is calculated in a straightforward way from the formula:

$$L_p(\omega) = I - \alpha(\omega)A_1 - \alpha^2(\omega)A_2 - \ldots - \alpha^p(\omega)A_p \tag{3.1.6}$$

$$H_p(\omega) = L^{-1}(\omega) \tag{3.1.7}$$

where p is the order of the model.

3.1.3 Spectrum

Taking the Fourier transform of both sides of 3.1.2 and averaging, we obtain the Spectrum of Y as the top-left $(k \times k)$ sub-block of the matrix function:

$$\mathsf{S}_\mathsf{X}(\omega) = -\mathsf{G} + (\mathsf{I} - \alpha(\omega)\mathsf{A})^{-1}\mathsf{G} + \mathsf{G}(\mathsf{I} - \alpha^*(\omega)\mathsf{A}^T)^{-1} \tag{3.1.8}$$

$$= -\mathsf{G} + \mathsf{H}(\omega)\mathsf{G} + \mathsf{G}\mathsf{H}^*(\omega) \tag{3.1.9}$$

where A is the extended coefficient matrix of 3.1.2, G is the covariance matrix (autocovariance at lag 0) of the extended vector X and $\mathsf{H}(\omega)$ is the corresponding Transfer function of the extended model 3.1.2. To find the top-left $(k \times k)$ sub-block in 3.1.8, we solve the resulting block-matrix equations, which after some algebraic simplification, gives:

$$S_Y(\omega) = -G_0 + T(\omega) + T^*(\omega) \tag{3.1.10}$$

$$\text{with} \qquad T(\omega) = H_p(\omega)\left\{ G_0 - \sum_{j=1}^{p-1} \alpha^{-j}(\omega)(L_j(\omega) - L_p(\omega))G_j \right\} \tag{3.1.11}$$

where G_j is the autocovariance of Y at lag j, and $L_j(\omega)$ is the expression 3.1.6 whose inverse is the Transfer Function $H_j(\omega)$ for an order j model.

3.2 Sub-models

Given the VAR model for the full set of variables and some given set of lags $\mathcal{L} = \{1, 2, \ldots, p\}$, where p is the order of the model, we now describe how to compute the VAR submodel for a subset of the variables and a subset of the lags. More precisely, we would like to compute the VAR model as if all the "data" is to be explained by only the specified subset of the original set variables and the specified subset of the original set of lags. Denote the original set of variables by:

$$W = \{w^{(1)}, w^{(2)}, \ldots, w^{(k)}\}^T$$

We write the full VAR model, with assumptions as in Main [2]:

$$W_t - \sum_{i \in \mathcal{L}} A_i W_{t-i} = \epsilon_t \tag{3.2.1}$$

Let X denote the subset of variables for which we require the sub-model. Let Z denote the complement of X in W. Without loss of generality we may assume that for some $s < k$,

$$X = \{w^{(1)}, w^{(2)}, \ldots, w^{(s)}\} \tag{3.2.2}$$
$$Z = \{w^{(s+1)}, \ldots, w^{(p)}\} \tag{3.2.3}$$
$$W = [X, Z] \tag{3.2.4}$$

(Otherwise permute the variables appropriately).

Partition the noise terms ϵ_t and each coefficient matrix A_i of the original model as:

$$A_i = \begin{bmatrix} B_i^{xx} & B_i^{xz} \\ B_i^{zx} & B_i^{zz} \end{bmatrix} \tag{3.2.5}$$
$$\epsilon_t = \begin{bmatrix} \mu_t^x & \mu_t^z \end{bmatrix} \tag{3.2.6}$$

where B_i^{xx} is a $(s \times s)$ coefficient matrix at lag i pertaining only to X, and μ_t^x is the noise pertaining only to to X.

Also denote by $\mathcal{J}$ the subset of the original lags ($\mathcal{L}$) over which the fit is required, and let $\mathcal{M}$ denote the complement of $\mathcal{J}$ in $\mathcal{L}$. We can then write the equations for X as:

$$X_t - \sum_{i \in \mathcal{J}} B_i^{xx} X_{t-i} = \sum_{i \in \mathcal{M}} B_i^{xx} X_{t-i} + \sum_{i \in \mathcal{L}} B_i^{xy} Z_{t-i} + \mu_t^x \tag{3.2.7}$$

and for the purpose of fitting the submodel, we write:

$$X_t - \sum_{i \in \mathcal{J}} C_i X_{t-i} = \nu_t \tag{3.2.8}$$

The right hand side of (3.2.8) is the 'noise' which is, by hypothesis, uncorrelated with $X(t-i)$ for $i \geq 1$. We therefore have to "fit" the coefficients C_i to the model represented by (3.2.8). The autocovariance function (which can be thought of as the autocovariance of the "data") can be calculated directly from the coefficients of the original model. We can therefore use the Yule-Walker equations for the original model (restricted to the components in X) to fit coefficients C_i in (3.2.8) and then compute the new noise term ν_t. From equations (3.2.7) and (3.2.8), this noise term is:

$$\nu_t = \sum_{i \in \mathcal{J}} (B_i^{xx} - C_i) X_{t-i} + \sum_{i \in \mathcal{M}} B_i^{xx} X_{t-i} + \sum_{i \in \mathcal{L}} B_i^{xy} Z_{t-i} + \mu_t^x \tag{3.2.9}$$
$$= \sum_{i \in \mathcal{L}} (B_i^{xx} - C_i \mathbb{1}_i^{\mathcal{J}}) X_{t-i} + \sum_{i \in \mathcal{L}} B_i^{xy} Z_{t-i} + \mu_t^x \tag{3.2.10}$$
$$= \sum_{i \in \mathcal{L}} F_i^x W_{t-i} + \mu_t^x \tag{3.2.11}$$

where $F_i^x = [B_i^{xx} - C_i \mathbb{1}_i^{\mathcal{J}}, B_i^{xy}]$, and $\mathbb{1}^{\mathcal{J}}$ is the indicator function of $\mathcal{J}$ on $\mathcal{L}$.

As per the original model hypothesis, the W_{t-i}, for $i \geq 1$, are not correlated with μ_t^x. Using this fact, and after some algebraic simplification, the covariance matrix of ν_t is seen to be:

$$\Sigma_\nu = \mathsf{F}^{\mathsf{x}} \mathsf{G} \mathsf{F}^{\mathsf{x}\mathsf{T}} + \Sigma_{\mu^{\mathsf{x}}} \tag{3.2.12}$$

where:

$$\mathsf{F}^{\mathsf{x}} = \left[F_1^x, F_2^x, \ldots, F_p^x\right] \tag{3.2.13}$$

$$\mathsf{G} = \begin{bmatrix} G_0 & G_1 & G_2 & \ldots & G_{p-1} \\ G_{-1} & G_0 & G_1 & \ldots & G_{p-2} \\ G_{-2} & G_{-1} & G_0 & \ldots & G_{p-3} \\ \ldots \\ \ldots \\ G_{-p+2} & G_{-p+3} & G_{-p+4} & \ldots & G_1 \\ G_{-p+1} & G_{-p+2} & G_{-p+3} & \ldots & G_0 \end{bmatrix} \tag{3.2.14}$$

where G_h is the autocovariance of the original full set of variables Wat lag h and Σ_{μ^x} is the covariance matrix of $\mu^{(x)}$.

This allows us to calculate the coefficients C_i and the new VAR model. Note that it involves no further calculation other than solving the Yule-Walker equations restricted to the subset X, and computing the new noise term ν_t. For the left hand side matrix of the Yule-Walker equations, a one time computation of the autocovariances of the full model as per 3.1.4 suffices for all submodels. We discuss an efficient implementation of the subfit computations above in section 5.

Using the procedures outlined in sections 3.1 and 3.2, we are now ready to calculate the Granger Causality strengths and spectra as below. For more details on how to obtain the Granger Causality formulas below see [6, pg. 451-474]. For insight into the reasoning and rationale behind the definitions below, see [7].

3.3 Granger Causalities

Define the Lag operator $\mathcal{L}$ by $\mathcal{L}(X_t) = X_{t-1}$. In the remainder of this section and the next we will find it convenient to write VAR equations Main [2] in operator form:

$$[I - A(\mathcal{L})].Y_t = \epsilon_t \tag{3.3.1}$$

Here $A(\mathcal{L}) = A_1.\mathcal{L}^1 + A_2.\mathcal{L}^2 + \ldots + A_p.\mathcal{L}^p$.

3.3.1 Time Domain (GC strengths)

Let W be stochastic process, that can be represented under the same general conditions as in equation Main [2]. Suppose that W is composed of two sub-processes X and Y with $W = [X, Y]$. We can then represent W as:

$$X_t = \sum_{i=1}^{p} B_i^{xx} X_{t-i} + \sum_{i=1}^{p} B_i^{xy} Y_{t-i} + \epsilon_t^x$$
$$Y_t = \sum_{i=1}^{p} B_i^{yx} X_{t-i} + \sum_{i=1}^{p} B_i^{yy} Y_{t-i} + \epsilon_t^y$$

We write these in operator form as in 3.3.1:

$$\left(\begin{bmatrix} I^x & 0 \\ 0 & I^y \end{bmatrix} - \begin{bmatrix} B^{xx}(\mathcal{L}) & B^{xy}(\mathcal{L}) \\ B^{yx}(\mathcal{L}) & B^{yy}(\mathcal{L}) \end{bmatrix}\right) \cdot \begin{bmatrix} X_t \\ Y_t \end{bmatrix} = \begin{bmatrix} \epsilon_t^x \\ \epsilon_t^y \end{bmatrix} \tag{3.3.2}$$

with the error covariance matrix for ϵ given by:

$$\mathrm{T} = \begin{bmatrix} \mathrm{T}^{xx} & \mathrm{T}^{xy} \\ \mathrm{T}^{xy'} & \mathrm{T}^{yy} \end{bmatrix} \tag{3.3.3}$$

In addition, assume that the two sub-processes, X and Y, individually satisfy the same general assumptions as in Main [2] so that we can write them as:

$$\left(\begin{bmatrix} I^x & 0 \\ 0 & I^y \end{bmatrix} - \begin{bmatrix} A^x(\mathcal{L}) & 0 \\ 0 & A^y(\mathcal{L}) \end{bmatrix}\right) \cdot \begin{bmatrix} X_t \\ Y_t \end{bmatrix} = \begin{bmatrix} \delta_t^x \\ \delta_t^y \end{bmatrix}$$

with the error covariance matrix for δ given by:

$$\Sigma = \begin{bmatrix} \Sigma^x & 0 \\ 0 & \Sigma^y \end{bmatrix}$$

In the time domain, the Granger Causality $F_{Y \to X}$ from Y to X is defined by:

$$F_{Y \to X} = \ln \left[\frac{|\Sigma^x|}{|\mathrm{T}^{xx}|} \right] \tag{3.3.4}$$

where $|.|$ denotes the determinant. The Granger Causality from X to Y is defined analogously. Having fitted a VAR model to the full set of data, the GC strengths can be computed using the above formulas and the formulas in section 3.2 for the coefficients and noise covariance of the sub-fitted models.

3.3.2 Frequency Domain (GC spectrum)

First left multiply both sides of equation (3.3.2) by the normalizing matrix:

$$\begin{bmatrix} I^x & 0 \\ -\mathrm{T}^{xy'} {\mathrm{T}^{yy}}^{-1} & I^y \end{bmatrix} \tag{3.3.5}$$

The resulting equations:

$$\left(\begin{bmatrix} I^x & 0 \\ \tilde{B}_0^{yx} & I^y \end{bmatrix} - \begin{bmatrix} B^{xx}(\mathcal{L}) & B^{xy}(\mathcal{L}) \\ \tilde{B}^{yx}(\mathcal{L}) & \tilde{B}^{yy}(\mathcal{L}) \end{bmatrix}\right) \cdot \begin{bmatrix} X_t \\ Y_t \end{bmatrix} = \begin{bmatrix} \tilde{\epsilon}_t^x \\ \tilde{\epsilon}_t^y \end{bmatrix} \tag{3.3.6}$$

include a projection of Y onto not only the past, but also the present of X, in such a way that the cross covariance between the X and Y blocks of the new noise terms vanishes:

$$\mathsf{Cov}(\tilde{\epsilon}) = \begin{bmatrix} \mathrm{T}^{xx} & 0 \\ 0 & \tilde{\mathrm{T}}^{yy} \end{bmatrix} \tag{3.3.7}$$

Because of this independence, the spectrum $S^{(xx)}$ of X can be decomposed as:

$$\widetilde{H}^{xx}(\omega)\mathrm{T}^{xx}\widetilde{H}^{xx*}(\omega) + \widetilde{H}^{xy}(\omega)\tilde{\mathrm{T}}^{yy}\widetilde{H}^{xy*}(\omega)$$

where $\widetilde{H}^{xx}(\omega)$ and $\widetilde{H}^{xy}(\omega)$ are blocks of the transfer function (3.1.6) of the normalized operator (3.3.6). $\widetilde{H}^{xx}(\omega)$ is related to the original X block transfer function by:

$$\widetilde{H}^{xx}(\omega) = H^{xx}(\omega) + H^{xy}(\omega)\mathrm{T}^{xy}\{\mathrm{T}^{xx}\}^{-1} \tag{3.3.8}$$

The Granger Causality, $f_{Y \to X}(\omega)$, from Y to X at the frequency ω is then defined by:

$$f_{Y \to X}(\omega) = \ln \left(\frac{|S^{(xx)}|}{|\widetilde{H}^{xx}(\omega)\mathrm{T}^{xx}\widetilde{H}^{xx*}(\omega)|} \right) \tag{3.3.9}$$

As in the case of the GC strengths, once a VAR model is fit to the full set of data, the GC spectrum can be computed using the above formulas and the formulas in section 3.2 for the coefficients and noise covariance of the sub-fitted models.

3.4 Conditional Granger Causalities

For three or more time series, one can take pairs of time series components and compute the Granger Causality for each of these pairs. This is called a pairwise analysis, but it has inherent limitations. We do not know if Y is genuinely driven by X or only appears to be driven by X due to the presence of an intermediary Z which transmits the influence to Y. To determine whether a causal influence from variable X to Y is partially or wholly mediated by some other variable Z, we need the concept of conditional Granger Causality, both in the time and in the spectral domains. We briefly mention the relevant equations here. For more details on how to obtain these formulas, see [8] or [9, pg. 228–237] and the references therein. For insight into the reasoning and rationale behind the definitions below, see [10].

3.4.1 Time Domain (conditional GC strengths)

Consider three stochastic processes X_t, Y_t and Z_t. Suppose that a pairwise analysis shows Granger Causality from Y to X. To determine whether this influence is partially or wholly due to the presence of Z, first write the VAR model equations for the processes X_t and Z_t taken together:

$$\left(\begin{bmatrix} I^x & 0 \\ 0 & I^z \end{bmatrix} - \begin{bmatrix} B^{xx}(\mathcal{L}) & B^{xz}(\mathcal{L}) \\ B^{zx}(\mathcal{L}) & B^{zz}(\mathcal{L}) \end{bmatrix}\right) \cdot \begin{bmatrix} X_t \\ Z_t \end{bmatrix} = \begin{bmatrix} \epsilon_t^x \\ \epsilon_t^z \end{bmatrix} \tag{3.4.1}$$

Denote the covariance matrix of the noise terms by:

$$\mathrm{T} = \begin{bmatrix} \mathrm{T}^{xx} & \mathrm{T}^{xz} \\ \mathrm{T}^{xz'} & \mathrm{T}^{zz} \end{bmatrix} \tag{3.4.2}$$

Next write the equations for all three processes X, Y and Z taken together:

$$\left(\begin{bmatrix} I^x & 0 & 0 \\ 0 & I^y & 0 \\ 0 & 0 & I^z \end{bmatrix} - \begin{bmatrix} C^{xx}(\mathcal{L}) & C^{xy}(\mathcal{L}) & C^{xz}(\mathcal{L}) \\ C^{yx}(\mathcal{L}) & C^{yy}(\mathcal{L}) & C^{yz}(\mathcal{L}) \\ C^{zx}(\mathcal{L}) & C^{zy}(\mathcal{L}) & C^{zz}(\mathcal{L}) \end{bmatrix}\right) \cdot \begin{bmatrix} X_t \\ Y_t \\ Z_t \end{bmatrix} = \begin{bmatrix} \phi_t^x \\ \phi_t^y \\ \phi_t^z \end{bmatrix} \tag{3.4.3}$$

and denote the matrix of error covariance terms by:

$$\Upsilon = \begin{bmatrix} \Upsilon^{xx} & \Upsilon^{xy} & \Upsilon^{xz} \\ \Upsilon^{yx} & \Upsilon^{yy} & \Upsilon^{yz} \\ \Upsilon^{zx} & \Upsilon^{zy} & \Upsilon^{zz} \end{bmatrix} \tag{3.4.4}$$

where $\Upsilon^{yx} = \Upsilon^{xy'}$, $\Upsilon^{zx} = \Upsilon^{xz'}$ and $\Upsilon^{zy} = \Upsilon^{yz'}$.

Then the Granger Causality from Y to X, conditional on Z is defined to be:

$$F_{Y \to X|Z} = ln \left[\frac{|\Upsilon^{xx}|}{|\mathrm{T}^{xx}|}\right] \tag{3.4.5}$$

The Granger Causality from X to Y is defined analogously. As in the case of the pairwise GC strengths, once a VAR model is fit to the full set of data, the conditional GC strengths can be computed using the above formulas and the formulas in section 3.2 for the coefficients and noise covariance of the sub-fitted models.

3.4.2 Frequency Domain (conditional GC spectrum)

The computation of the Conditional Granger Causality in the frequency domain involves a series of steps. In what follows I_x, I_y and I_z denote identity matrices of the appropriate block size.

As in the case of the pairwise GC spectrum, first multiply both sides of equation (3.4.1) by the normalizing matrix:

$$P_0 = \begin{bmatrix} I^x & 0 \\ -\mathrm{T}^{xz'}\mathrm{T}^{xx^{-1}} & I^z \end{bmatrix}$$

This projects the variable Z onto the past as well as the present of X in a way that absorbs the cross covariance between the noise terms of X and Y. Therefore, the resulting equations have independent noise covariance:

$$\left(\begin{bmatrix} I^x & 0 \\ \tilde{B}_0^{zx} & I^z \end{bmatrix} - \begin{bmatrix} B^{xx}(\mathcal{L}) & B^{xz}(\mathcal{L}) \\ \tilde{B}^{zx}(\mathcal{L}) & \tilde{B}^{zz}(\mathcal{L}) \end{bmatrix}\right) \cdot \begin{bmatrix} X_t \\ Z_t \end{bmatrix} = \begin{bmatrix} \tilde{\epsilon}_t^x \\ \tilde{\epsilon}_t^z \end{bmatrix} \tag{3.4.6}$$

$$\mathsf{Cov}(\tilde{\epsilon}) = \begin{bmatrix} \Upsilon^{xx} & 0 \\ 0 & \tilde{\Upsilon}^{zz} \end{bmatrix} \tag{3.4.7}$$

Similarly multiply both sides of equation (3.4.3) by P [$= P_2.P_1$] where

$$P_1 = \begin{bmatrix} I^x & 0 & 0 \\ -\Upsilon^{yx}{\Upsilon^{xx}}^{-1} & I^y & 0 \\ -\Upsilon^{zx}{\Upsilon^{xx}}^{-1} & 0 & I^z \end{bmatrix},$$

$$P_2 = \begin{bmatrix} I^x & 0 & 0 \\ 0 & I^y & 0 \\ 0 & -(\Upsilon^{zy} - \Upsilon^{zx}{\Upsilon^{xx}}^{-1}\Upsilon^{xy})(\Upsilon^{yy} - \Upsilon^{yx}{\Upsilon^{xx}}^{-1}\Upsilon^{xy})^{-1} & I^z \end{bmatrix}$$

Again, this projects the variable Y onto the past and present of X, and the variable Z onto the past and present of both X and Y, in such a way that the resulting equations have independent noise covariance:

$$\left(\begin{bmatrix} I^x & 0 & 0 \\ \tilde{C}_0^{yx} & I^y & 0 \\ \tilde{C}_0^{zx} & \tilde{C}_0^{zy} & I^z \end{bmatrix} - \begin{bmatrix} \tilde{C}^{xx}(\mathcal{L}) & \tilde{C}^{xy}(\mathcal{L}) & \tilde{C}^{xz}(\mathcal{L}) \\ \tilde{C}^{yx}(\mathcal{L}) & \tilde{C}^{yy}(\mathcal{L}) & \tilde{C}^{yz}(\mathcal{L}) \\ \tilde{C}^{zx}(\mathcal{L}) & \tilde{C}^{zy}(\mathcal{L}) & \tilde{C}^{zz}(\mathcal{L}) \end{bmatrix}\right) \cdot \begin{bmatrix} X_t \\ Y_t \\ Z_t \end{bmatrix} = \begin{bmatrix} \tilde{\phi}_t^x \\ \tilde{\phi}_t^y \\ \tilde{\phi}_t^z \end{bmatrix} \tag{3.4.8}$$

$$\mathsf{Cov}(\tilde{\phi}) = \begin{bmatrix} \tilde{\Upsilon}^{xx} & 0 & 0 \\ 0 & \tilde{\Upsilon}^{yy} & 0 \\ 0 & 0 & \tilde{\Upsilon}^{zz} \end{bmatrix} \tag{3.4.9}$$

Now let $\tilde{H}_b(\omega)$ and $\tilde{H}_c(\omega)$, respectively, denote the transfer functions, as defined in section 3, of the operators on the left hand sides of the equations (3.4.6) and (3.4.8). Then define the matrix $Q(\omega)$ by:

$$\begin{bmatrix} Q^{xx} & Q^{xy} & Q^{xz} \\ Q^{yx} & Q^{yy} & Q^{yz} \\ Q^{zx} & Q^{zy} & Q^{zz} \end{bmatrix}(\omega) = \begin{bmatrix} \tilde{H_b}^{xx}(\omega) & 0 & \tilde{H_b}^{xz}(\omega) \\ 0 & I^y & 0 \\ \tilde{H_b}^{zx}(\omega) & 0 & \tilde{H_b}^{zz}(\omega) \end{bmatrix}^{-1} \begin{bmatrix} \tilde{H_c}^{xx} & \tilde{H_c}^{xy} & \tilde{H_c}^{xz} \\ \tilde{H_c}^{yx} & \tilde{H_c}^{yy} & \tilde{H_c}^{yz} \\ \tilde{H_c}^{zx} & \tilde{H_c}^{zy} & \tilde{H_c}^{zz} \end{bmatrix}(\omega) \tag{3.4.10}$$

The Conditional Granger Causality Spectrum from Y to X conditional on Z is then defined as:

$$f_{Y \to X|Z} = ln\left[\frac{|\Upsilon^{xx}|}{|Q^{xx}(\omega)\tilde{\Upsilon}^{xx}Q^{xx*}(\omega)|}\right] \tag{3.4.11}$$

For more details and insight into these formulas, see [10]. The Conditional Granger Causality Spectrum from X to Y conditional on Z is defined analogously. As in the case of the pairwise GC spectrum, once a VAR model is fit to the full set of data, the conditional GC spectrum can be computed using the above formulas and the formulas in section 3.2 for the coefficients and noise covariance of the sub-fitted models.

3.5 Consistency between GC strengths and spectrum

For the pairwise granger causalities and spectrum , the consistency condition which ensures the equality of $f_{Y \to X}$ and $\int_\omega f_{Y \to X}(\omega)$ can be stated as follows:

The pairwise GC spectrum is consistent with the GC strengths only if the VAR operator $\tilde{B}^{yy}$ in equations (3.3.6) is stable. If $\tilde{B}^{yy}$ is not stable, then $\int_\omega f_{Y\to X}(\omega) < f_{Y\to X}$.

For conditional Granger Causality, the corresponding condition is:

Let $\mathcal{L}_Q^{yz}$ be the YZ block of the VAR operator whose transfer function is the left hand side Q of equation (3.4.10). Then the conditional GC spectrum is consistent with the conditional GC strengths only if $\mathcal{L}_Q^{yz}$ is stable, otherwise $\int_\omega f_{Y\to X|Z}(\omega) < f_{Y\to X|Z}$.

For the granger causalities in the opposite directions, reverse the roles of X and Y everywhere. For proofs of these see [7] and [10] respectively.

Note that to compute all pairs of granger causalities in a multivariable model, one would use the sub-fitting procedure described in section 3.2 on each pair of variables (or each pair of blocks as the case may be) and then use the formulas for the granger causalities above. For the conditional granger causalities, to get $f_{Y\to X|Z}$, subfits must be performed for the YZ and XYZ blocks. Thus the GC consistency condition must be checked separately for each pair of interacting blocks.

3.5.1 GC consistency condition for random models

Our numerical tests (on pairwise GC) show that random dense models, are unlikely to satisfy the GC consistency condition for all interactions. On generating a large number of random dense models, we found that the probability that a random dense model satisfies the consistency condition, decreases as the model size gets larger and in general this probability is a very small but finite value. On the other hand, similar tests on sparse models show that random sparse models (of the sparsity levels that concern us here) satisfy the consistency condition with high probability.

The practical implication of this is that for sufficiently dense VAR models, there will be large discrepancies between the integrated GC spectrum and the GC strengths for those interactions that do not satisfy the GC consistency condition. The discrepancies become particularly egregious as the coefficient density becomes high.

4 Additional performance measures

We include a few more performance measures here in addition to the ones in section Main [3].

The reference models against which the tests were made were all checked for the pairwise consistency condition stated in section 3.5] and they were, as expected, all GC-consistent with respect to both Pairwise and Conditional Granger Causalities. In general sparse models appear to have a high probability of passing the GC consistency test, while dense models have a fairly high probabilty of failing it for at least some interactions.

The models obtained via *SVARGS+CGC* were all stable (or required a very small deflation ($< 1\%$) of the coefficient size to make them stable) and all satisfied the GC consistency condition for Pairwise GC. We did not check the theoretical consistency condition for the *conditional* GC, because it would have involved approximating a theoretically infinite order VAR operator from its transfer function. However, the normalized difference between the Conditional GC strengths and sum of the Conditional GC spectrum was less than 0.05 in all cases which strongly suggests that they satisfy the Conditional GC consistency condition.

4.1 Error Measure Definitions

- **Percentage of unpredicted coefficients** $= 100 \times |L_r \bigcap L_f^c(M)|/|L_r$
- **Normalized RMS error in predicted coefficients** $= M^{-1/2}\|(r_i - f_i)/r_i, \quad i \in L_r\|_2$
- **Normalized bias in predicted coefficients** $= M^{-1}\sum_{i\in L_r}(r_i - f_i)/r_i$
- **Normalized RMS error in noise covariance** $= \|\Sigma_r - \Sigma_f\|_s/\|\Sigma_r\|_s$

- **Order/Lag error** $= \sum_{q\in Q_s} \phi_q/\|f_i^2, i \in L_r\|_2, \quad \phi_q = max_{f\in F_q}(|f|)$
- **Percent spurious relative to actual number of cofficients** $= 100 \times |L_f(M) \bigcap L_r^c|/|L_r|$
- **Normalized RMSE in CGC** $= 100 \times M^{-1/2}\|(r_{gi} - f_{gi})/r_{gi}, \quad i \in G_r\|_2$
- **Percent unpredicted CGC interactions** $= 100 \times |G_r \bigcap G_f^c(M)|/|G_r|$
- **Percent spurious CGC** $= 100 \times |G_f(M) \bigcap G_r^c|/|G_r|$

The symbols used above are defined as below:

- M: Number of non-zero coefficient/CGC (as per context) locations in the reference model.
- F_q: Matrix of fitted coefficients at lag q.
- G_r: Index set of the non-zero Conditional GC in the reference model.
- G_f: Index set of the M largest/most significant non-zero Conditional GC in the fitted model.
- L_r: Index set of the non-zero coeffs in reference model.
- L_f: Index set of the M largest non-zero coeffs in reference fitted model.
- r_i, f_i: Coefficient at location i in (reference, fitted) model.
- r_{gi}, f_{gi}: Conditional GC at location i in (reference, fitted) model.
- Σ_r, Σ_f: Noise covariance matrix of (reference, fitted) model.
- Q_s: Lags in fitted model not present in the reference model.
- $\|.\|_s$: entrywise 2-norm (frobenius norm) of a matrix.
- $\|.\|_2$ denotes the 2-norm of a vector.
- If U is a set, $|U|$ denotes the cardinality of U, U^c denoted the complement of U.
- If s is a scalar, $|s|$ is the absolute value of s.

4.2 Model Fit and Conditional GC Performance

Figures 3 , 2 and 4 show the performance of the SVARGS with respect to the coefficients and also with respect to the conditional Granger Causality.

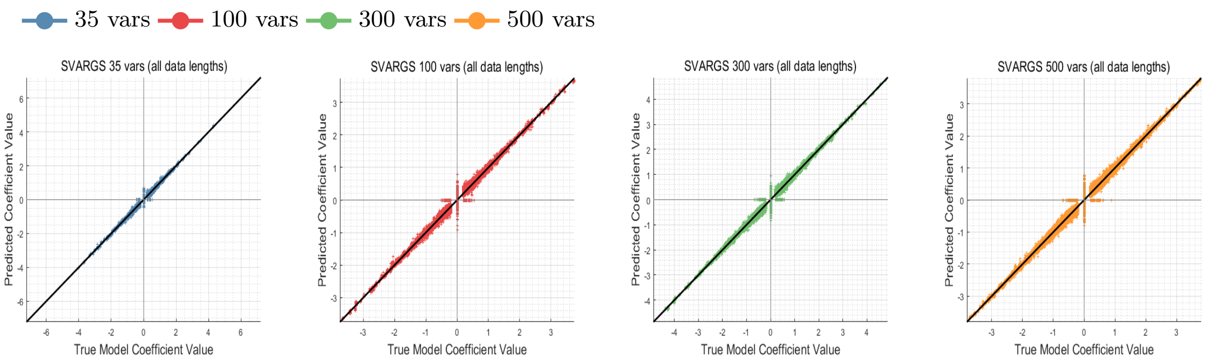

(a) 35 variables (b) 100 variables (c) 300 variables (d) 500 variables

Figure 1: **Coefficient Scatter: SVARGS**. The figures show a scatter plot of the actual coefficients vs. the coefficients predicted by SVARGS. For each of the four model sizes, the plots show the combined scatter over 50 random VAR models and 7 different data lengths $150, 200, 250, 300, 350, 400, 450$.

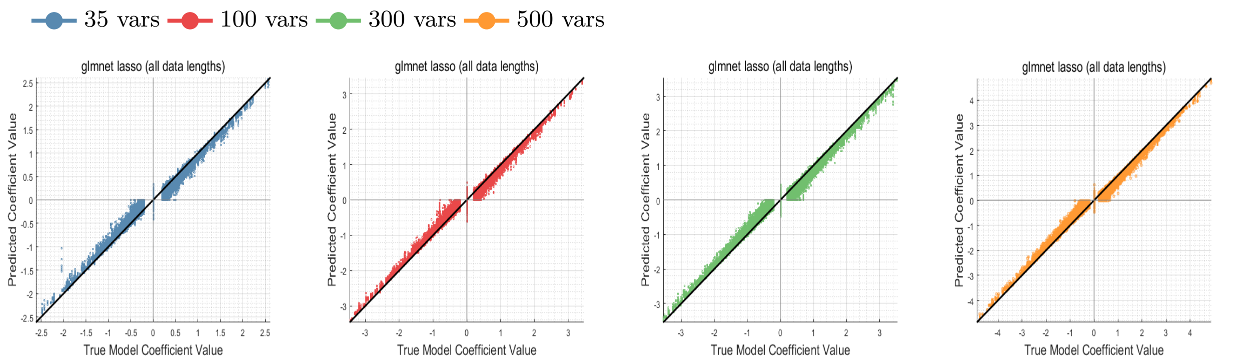

(a) 35 variables (b) 100 variables (c) 300 variables (d) 500 variables

Figure 2: **Coefficient Scatter: glmnet-lasso**. The figures show a scatter plot of the actual coefficients vs. the coefficients predicted by glmnet-lasso. For each of the four model sizes, the plots show the combined scatter over 50 random VAR models and 7 different data lengths 150, 200, 250, 300, 350, 400, 450.

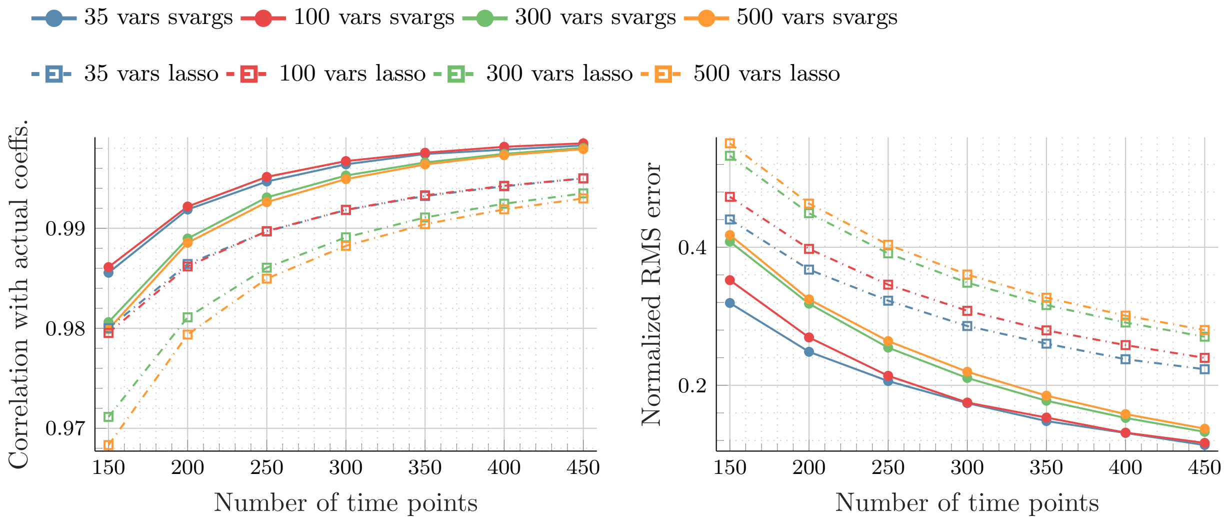

(a) Correlation with true coefficients. (b) Relative RMSE

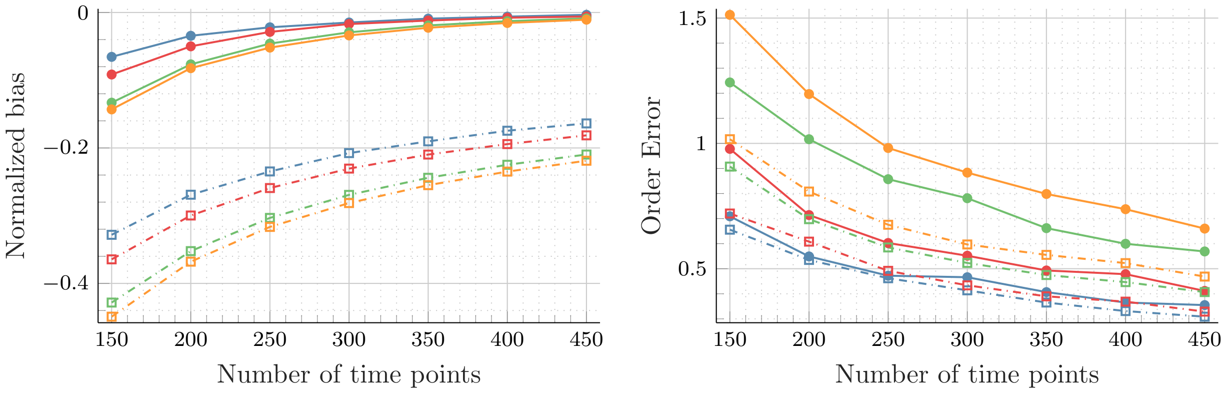

(c) Normalized Bias (d) Order Error

Figure 3: **Additional coefficient performance measures**. The figures show error measures for the coefficients computed by SVARGS as well as those computed by glmnet-lasso. For each variable length above, the results were averaged over 50 random VAR models and 7 different data lengths 150, 200, 250, 300, 350, 400, 450.

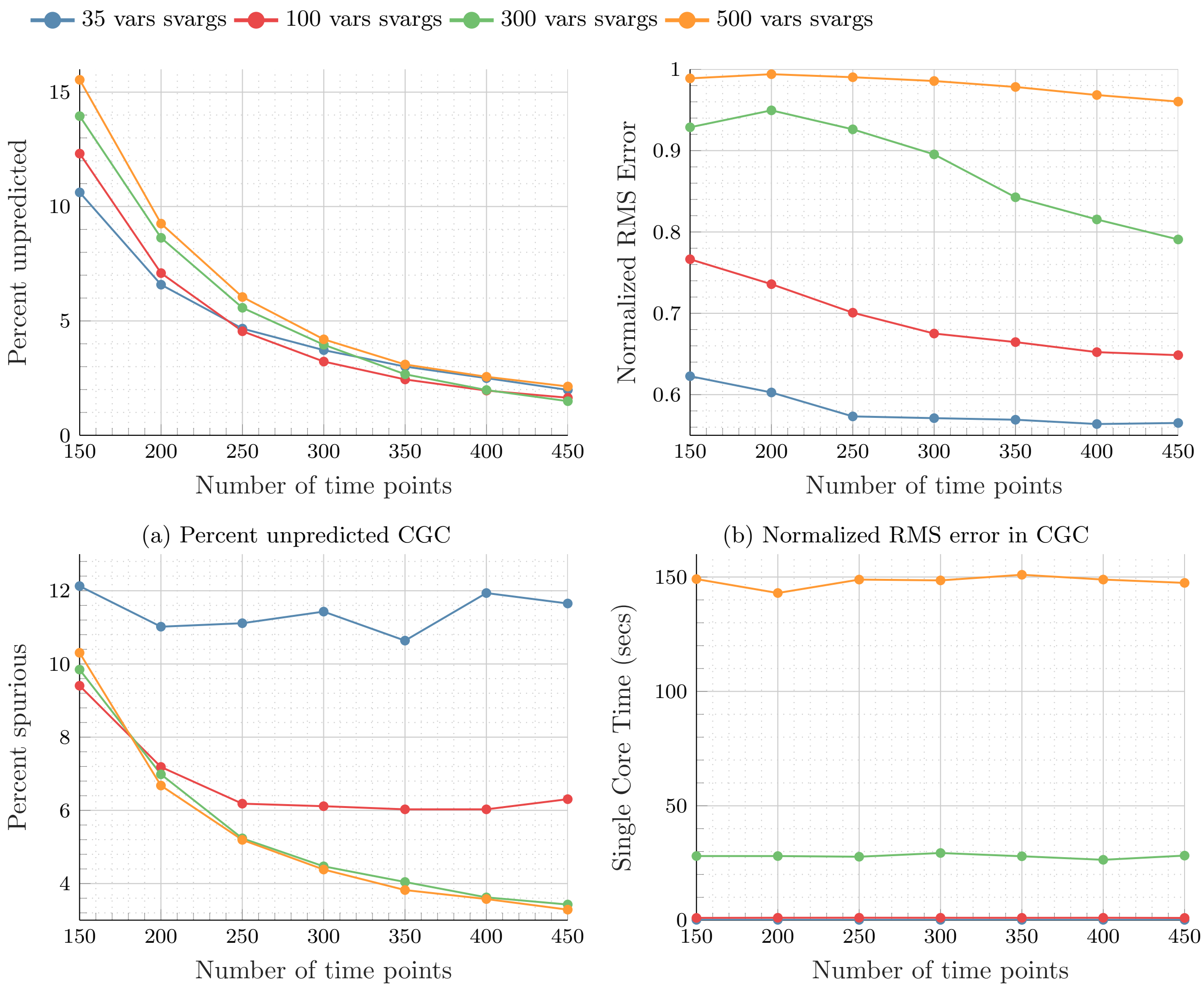

(a) Percent unpredicted CGC

(b) Normalized RMS error in CGC

(c) Percent Spurious CGC

(d) Single Core Seconds to compute CGC

Figure 4: **Conditional GC performance**. The figures show error measures for the Conditional GC computed by SVARGS+CGC. For each variable length above, the results were averaged over 50 random VAR models and 7 different data lengths 150, 200, 250, 300, 350, 400, 450.

4.3 Performance on Pairwise Granger Causality (35 variables)

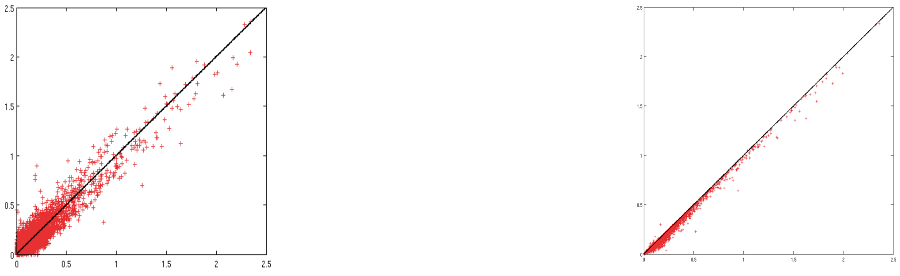

(a) True Strengths vs. Pred. Strengths

(b) Pred. Strengths vs. Summed Pred. Spectrum.

Figure 5: Pairwise GC Performance

4.4 Performance with Added Noise

We also computed VAR models from simulated data to which simulated external (measurement) noise had been added. The standard deviations of the simulated noise used were 0, 0.2, 0.4 and 0.6 of the standard deviations of the intrinsic noise. For each noise level, the methods were evaluated on 10 randomly generated VAR models. Figure 6 shows the performance of SVARGS in the presence of added (measurement) noise.

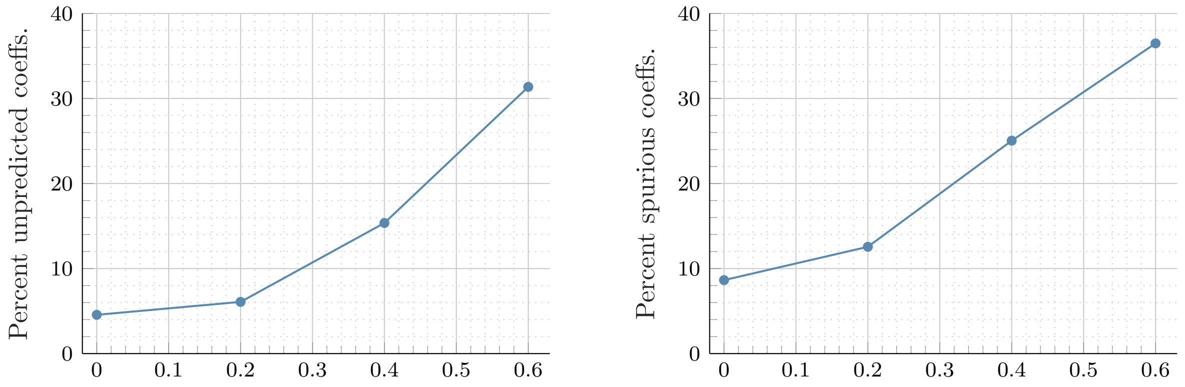

(a) Percent unpredicted, k = 35, N = 200

(b) Percent spurious, k = 35, N = 200

Figure 6: **Performance with added noise**: Plotted with respect to the relative size of the std. deviation of the added noise. The data length used was 200 and the results averaged over 10 samples.

5 Autocovariance and Granger Causality computation

5.1 Autocovariance computation

There is no convenient closed form expression for the autocovariances G_j in (3.1.4). For smaller systems ($<$ 200 variables or so) we can efficiently compute the G_j by setting up the coefficients in the matrix equations and determining, programmatically, the matrix corresponding to the linear equation to be solved. This is much faster than trying to directly use the equations for extended autocovariance. However for larger systems, this becomes inefficient. We then use an iterative solver to solve the extended autocovariance equations (3.1.3), which are in the form of *Discrete Lyapunov Equations*. The Matlab `dlyap` method can be used to solve these.

Another faster option is to use a power doubling method described in [11] modified by us to take into account the special structure of the matrix A. This is particularly suitable for sparse models and is much faster than `dlyap`. Both these methods were used successfully in our applications.

Another approach that works well for dense models is to diagonalize the block companion matrix A in the expression for the extended autocovariance in section 3.1.1, as $\mathsf{A} = \mathsf{P}\Lambda\mathsf{P}^{-1}$, where Λ is a diagonal matrix with terms l_i on the diagonal and P is the matrix of eigenvectors of A.

The equation (3.1.3) for the extended autocovariance then becomes:

$$\tilde{\mathsf{G}} = \Lambda^{\mathsf{T}}\tilde{\mathsf{G}}\Lambda + \tilde{\Sigma} \tag{5.1.1}$$

where $\tilde{\mathsf{G}} = \mathsf{P}^{-1}\mathsf{G}\mathsf{P}^{\mathsf{T}^{-1}}$ and $\tilde{\Sigma} = \mathsf{P}^{-1}\Sigma\mathsf{P}^{\mathsf{T}^{-1}}$. The matrix A must be non-defective upto numerical tolerance for this to work properly.

The equation (5.1.1) has the explicit solution:

$$\mathsf{G} = \mathsf{P}\mathsf{T}\mathsf{P}^{\mathsf{T}} \tag{5.1.2}$$

where $\mathsf{T}_{\mathsf{ij}} = \tilde{\Sigma}_{\mathsf{ij}}/(1 - \mathsf{l_i l j})$.

This works better for dense rather than sparse matrices since a dense A is much more likely to be non-defective. For defective A, a similar explicit solution can be found in terms of the jordan canononical form, but this is impractical to compute for all but the smallest matrices.

Note that the iterative methods for computing the autocovariance require that the VAR model be stable.

5.2 Efficient subfits for conditional Granger Causality computation

With reference to section 3.2, when computing conditional granger causalities, a "leave one block out" subfit needs to be performed for each of the blocks where the conditional Granger Causality is required. We compute the conditional Granger Causality between two variables (or two, typically small, blocks of variables) conditioned on all the remaining variables. More generally, this is the situation where for each subfit, the subset of variables X to be subfit contains most of the variables in the system W (meaning, in each case, the subset of variables Z to be left out is small in relation to W). In such cases the subfit can be computed quite efficiently, without having to invert a large autocovariance matrix each time to find C and without having to perform the large matrix multiplication given by equation (3.2.12). This is done by computing the differentials in the noise and the coefficients rather than the actual quantities themselves. We state this result in a proposition (refer to section 3.2 for notation):

Proposition 1 (Noise delta and Coefficient delta for VAR model subfit)**.**
Let G *be as in equation* (3.1.3)*. Denote the inverse of* G *by* Q*. Let* X *(with* k_x *variables) and* Z *(with* k_z *variables) be a partition of the variables into two blocks as in equations* (3.2.2)*. Let* $\mathsf{G_x}$ *be the* $(k_x \times k_x)$ *submatrix of* G *that has the lag-extended autocovariance terms of* X*. Let* g *be the* $(k_z \times k_x)$ *submatrix of* G *that has the lag-extended cross-covariance between* Z *and* X *so that:*

$$\mathsf{G} = \begin{bmatrix} \mathsf{G_x} & \mathsf{g} \\ \mathsf{g}^\mathsf{T} & \mathsf{g_z} \end{bmatrix} \quad \mathsf{Q} = \begin{bmatrix} \mathsf{Q_x} & \mathsf{q} \\ \mathsf{q}^\mathsf{T} & \mathsf{q_z} \end{bmatrix} \tag{5.2.1}$$

Let $\mathsf{B^{xx}} = vertcat(B_i^{xx})$*, let* $\mathsf{B^{zx}} = vertcat(B_i^{zx})$*, and* μ_x *be the noise, in the equations for the* X *variables as in equation* (3.2.5)*. Let* $\mathsf{C} = vertcat(C_i)$ *be the subfitted coefficients for* X*, and let* ν *be the sub-fitted noise as in equation* (3.2.8)*. Then the coefficient delta and noise delta are given, respectively by:*

$$\mathsf{C} - \mathsf{B^{xx}} = -(\mathsf{q}\mathsf{q_z}^{-1}\mathsf{B^{zx}})^\mathsf{T} \tag{5.2.2}$$

$$\Sigma_\nu - \Sigma_{\mu^x} = \mathsf{B^{zx}}^\mathsf{T}\mathsf{q_z}^{-1}\mathsf{B^{zx}} \tag{5.2.3}$$

The proof of (5.2.2) involves writing the equations for the coefficients in block form and is straightforward but tedious, so we omit it here.

5.2.1 Fast computation of conditional GC due to known sparse locations

The sizes of the variable blocks between which the conditional GC is to be computed are usually small. So the number of rows in $\mathsf{B^{zx}}$ and the number of columns in q are small (for atomic blocks, it is exactly the number of lags). Similarly q_z and g_z are just small square matrices. In addition, equation (5.2.2) also tells us that the conditional GC from block i to block j is only non-zero if, in the original equations, the coefficient block (j, i) is non-zero at some lag. This, further, greatly reduces the amount of work required. The only expensive computation is the one-time calculation of the inverse, Q, of G. Therefore, computing the coefficient and noise delta's using (5.2.2) is much faster than using the full equations in section 3.2.

If, on the other hand, the variable blocks are large, then the total number of GC values to be calculated is small (since the number of GC values is the square of the number of blocks), and so the of cost computing the new coefficients by inverting the covariance matrix corresponding to the complement of Z is not so relevant. In our implementation, we use equations (5.2.2) if the size of the variable block is less than $k/2$. Otherwise we directly use the equations is section 3.2. Here k is the total number of variables.

Note that computing the subfits require the computation of the autocovariance. As we remarked in the previous section, if an iterative method is used to compute the autocovariance, then the model must be stable.

6 SVARGS computation

A fast implementation of the main computations involved in the SVARGS method is described in the supplementary information of [5], under the section *efficient implementation of the algorithm*. The same implementation can be easily extended to variable blocks as long as the blocks are all of the same size

7 Measure and node (channel) preselection procedure

To reduce the number of measures/nodes, start with the full set of features $\mathcal{F}$ - this is the union of the features over all the feature blocks (Each feature block corresponds to one measure or one node). Set the initial loss $L_0 = 1$. We use the following iterative procedure briefly described below:

1. Randomly partition the dataset into an appropriate number K of folds.
2. Then, for each test fold, train a model using the features in $\mathcal{F}$ on the corresponding training fold using Adaboost-SAMME upto the required number of rounds.
3. Use the model obtained to classify the test fold and record the predictions. This step will involve calculating all the individual gains at every split on the test fold.
4. Since the individual gains are now calculated for each measure/node, average the gain of the corresponding feature block over all rounds.
5. Perform multiple runs of the adaboost-SAMME algorithm by repeating steps 1 to 4 for different K-fold partitions of the data and average the resulting gains and compute the aggregate loss $L_{\mathcal{F}}$ from the predictions over all the runs.
6. If $L_{\mathcal{F}} > L_0$, STOP.
7. Prune the feature blocks by clustering the set of gains into high gains and low gains. The heuristic used for doing this was as follows:
   (a) Sort the gain values in decreasing order resulting in a curve G.
   (b) Put the high outliers in G in the high gain cluster, the low outliers and zeros in the low gain cluster and remove all these from G.
   (c) Find the index x_0 where the slope of G is minimum. Impose a lower bound $x_0 = \max(M/2, x_0)$ on x_0 where M is the number of elements in G.
   (d) Add $G(x), x < x_0$ to the high gain cluster and put the rest in the low gain cluster.
8. Remove the feature blocks that have low gain. If no features have been removed, STOP.
9. Let $\mathcal{F}$ be the union of the features over the high gain feature blocks, let $L_0 = L_{\mathcal{F}}$ and return to step 1.

The features selected are then the ones remaining in $\mathcal{F}$.

8 Application to DEAP and ADHD Datasets

8.1 DEAP Preprocessing Steps

1. The recording was downsampled to 256 Hz using a mild anti-aliasing filter (EEGlab library ([12])).
2. The recording was passed to the PREP pipeline along with the boundary event markers described above. The PREP pipeline was setup to perform the following actions:
   - Remove line noise at 50 Hz in a filter-agnostic manner.
   - Compute the common average reference.
   - Detect and interpolate channels that were bad due to large deviations relative to the common average reference.

3. The cleaned channels from PREP were then epoched to remove inter-stimulus segments and fed to the *amica* program with the pca reduction option. The ICA sphereing and weight matrices returned from *amica* were used to compute the ICA components.
4. Next the ICA components were passed to the ADJUST toolbox. The ADJUST function identifies four types of artifacts in the independent components: Horizontal eye movements, vertical eye movements, eye blinks and "generic discontinuities". For each the subjects ADJUST identifed between 1 and 7 eye movement and blink components over the two batch recordings. If "generic discontinuities" were included as artifacts then ADJUST identified between 2 and 14 artifacted components for each subject.
5. ICA components identified as eye blinks, horizontal eye movements or vertical eye movements were removed. Generic discountinuities were ranked by importance and only as many removed as would cap the maximum number of number of channels removed to 10. The EEG channels were then reconstructed from the remaining non-artifacted ICA components using the inverse ICA matrices.
6. In view of the above, the maximum rank of the (channels $\times$ time) batch recordings was reduced from 32 to a number between 21 and 31. Since we needed a common set of channels for all the subjects, 21 common reconstructed EEG channels were chosen from the $10-20$ system in a way that excluded interpolated channels as well as maintained an even distribution of channel locations over the scalp. The channels chosen were: AF3, F7, F3, FC5, T7, CP5, P7, P3, Pz, O1, O2, P4, P8, CP6, T8, FC6, F4, F8, AF4, Fz and Cz.
7. As a final step, data values that were extreme outliers in each channel of each recording were interpolated. The outliers were detected by computing a robust mean and robust standard deviation for each channel and using a cutoff of 2.5 standard deviations.

8.2 DEAP feature preselection

To reduce the number of features we used the procedure described in section 7 above. This procedure was performed separately for different sets of measures where each set of measures had the same block size. This was done to prevent unequal block sizes from skewing the results. The order in which the each blockwise elimination was performed is as below:

1. Blocks generated by each node-wise measure.
2. Blocks generated by each channel, channel asymmetry and channel caudality.

By using the above procedure, some channels and channel differences were eliminated from consideration leaving the 15 channels, 9 left-right channel asymmetries and 6 channel caudalities from which the node-wise and edgwise features were constructed. The channels and channel differences (asymmetries and caudalities) used in the final classifier were as follows:

- Channels (15): AF3, F7, F3, T7, CP5, P7, P3, P4, P8, CP6, T8, F4, F8, AF4 and Cz.
- Left-right differences (9): AF3-AF4, F7-F8, F3-F4, FC5-FC6, T7-T8, CP-CP6, P7-P8, P3-P4, O1-O2
- Caudal differences (6): F7-P7, F3-P3, F4-P4, F8-P8, FC5-CP5, FC6-CP6.

For the time domain feature based classification, all the weighted 14 node-wise network measures were used while, interestingly, the binary network measures were eliminated (see table Main [1]).

For the CGC spectrum based classification based on band summed features, the measures eliminated depended on the particular emotion being classified. The measures that remained and that were used in the final classification for each emotion are listed below. (see table Main [1] for the full list of node-wise measures).

- **Valence**: Strengths-in and Strengths-out.
- **Arousal**: Clustering-Coefficients, Participation-in/out, Node-Betweenness-Centralities, Eigenvector-Centralities-in/out.
- **Liking**: Participation-in, Eigenvector-Centralities-out, Node-Betweenness-Centralities.
- **Dominance**: Participation-in, Eigenvector-Centralities-in/out, Node-Betweenness-Centralities.
- **Familiarity**: Strengths-in, LocalEfficiencies, ClusteringCoefficients, Participation-in/out, Eigenvector-Centralities-out, Node-Betweenness-Centralities.

In addition, to reduce the large number of network edges and coefficients (**CO**, **NE-time** and **NE-spect**, many of them zeros), the following general procedure was adopted: For any such coefficient or CGC matrix, the top 10% of entries by magnitude were first chosen. Then only those entries (features) in each matrix were chosen that had had a non-zero value in at least m data entries (videos) where m was the smallest number that was significant at a p-value of 0.01 under the hypothesis that the non-zero values were distributed randomly among the features. The value for m is easily computed since it would then follow a binomial $(N, 0.1)$ distribution where N is the number of data instances.

8.3 DEAP classification performance for different feature groups

Table 1: Binary cross-validated classification accuracy on DEAP data set, Time Domain. For each emotion, the first row shows the proportion of the majority class. The second row shows the accuracy obtained by Koelstra et al ([13]). The remaining rows show the accuracies for each of the feature group combinations.

Method	valence	arousal	liking	dominance	familiarity
MAJ. CLS	56.56	58.91	66.95	62.11	56.64
KOELSTRA ET AL	57.60	62.00	55.40	NaN	NaN
NS-time+NN-time	72.72 (0.61)	66.31 (0.55)	71.04 (0.48)	69.66 (0.56)	68.91 (0.50)
NS-time+NE-time	73.64 (0.58)	66.93 (0.50)	70.33 (0.56)	70.37 (0.50)	69.72 (0.52)
NS-time+NE-time+CO	74.05 (0.53)	66.12 (0.31)	69.89 (0.39)	70.33 (0.62)	69.50 (0.65)
NS-time+NE-time+NN-time	73.64 (0.47)	66.87 (0.66)	71.04 (0.79)	70.21 (0.60)	69.40 (0.52)
NS-time+NE-time+NN-time+CO	74.76 (0.53)	66.47 (0.74)	70.92 (0.43)	70.41 (0.55)	69.70 (0.40)
NS-time+CO	73.72 (0.47)	66.24 (0.53)	69.64 (0.34)	70.31 (0.62)	69.38 (0.67)
NS-time+NE-time+NN-time+CO+PERI	75.13 (0.64)	66.55 (0.66)	69.75 (0.61)	70.60 (0.55)	69.88 (0.42)

Table 2: Binary cross-validated classification accuracy on DEAP data set, Spectral. For each emotion, the first row shows the proportion of the majority class. The second row shows the accuracy obtained by Koelstra et al ([13]). The remaining rows show the accuracies for each of the feature group combinations.

Method	valence	arousal	liking	dominance	familiarity
MAJ. CLS	56.56	58.91	66.95	62.11	56.64
KOELSTRA ET AL	57.60	62.00	55.40	NaN	NaN
NS-spect+NN-spect	74.19 (0.38)	66.06 (0.34)	68.86 (0.42)	69.62 (0.37)	69.05 (0.55)
NS-spect+NE-spect	75.15 (0.52)	67.46 (0.55)	70.40 (0.45)	72.01 (0.41)	69.94 (0.67)
NS-spect+NE-spect+CO	75.25 (0.49)	66.81 (0.59)	70.16 (0.53)	71.76 (0.76)	69.60 (0.69)
NS-spect+NN-spect+NE-spect	75.38 (0.52)	66.47 (0.35)	70.16 (0.59)	71.44 (0.82)	70.06 (0.40)
NS-spect+NN-spect+NE-spect+CO	75.55 (0.55)	66.38 (0.43)	69.95 (0.56)	70.96 (0.53)	69.57 (0.55)
NS-spect+NE-spect+PERI	75.71 (0.57)	67.82 (0.53)	70.47 (0.63)	71.60 (0.32)	69.73 (0.50)

Table 3: Feature Counts for different combinations of feature groups, Time Domain

Method	valence	arousal	liking	dominance	familiarity
NS-time+NN-time	555	555	555	555	555
NS-time+NE-time	101	101	101	101	101
NS-time+NE-time+CO	541	541	541	541	541
NS-time+NE-time+NN-time	647	647	647	647	647
NS-time+NE-time+NN-time+CO	1087	1087	1087	1087	1087
NS-time+CO	449	449	449	449	449
NS-time+NE-time+NN-time+CO+PERI	1164	1164	1164	1164	1164

Table 4: Feature Counts for different combinations of feature groups, Spectral

Method	valence	arousal	liking	dominance	familiarity
NS-spect+NN-spect	603	1695	876	1149	1968
NS-spect+NE-spect	718	718	718	718	718
NS-spect+NE-spect+CO	1158	1158	1158	1158	1158
NS-spect+NE-spect+NN-spect	1264	2356	1537	1810	2629
NS-spect+NE-spect+NN-spect+CO	1704	2796	1977	2250	3069
NS-spect+NE-spect+PERI	795	795	795	795	795

References

[1] Priestley M B. *Time Series and Spectral Analysis*, volume 1. Academic Press, 1981.

[2] Wilks S S. The large-sample distribution of the likelihood ratio for testing composite hypotheses. *The Annals of Mathematical Statistics*, 9(1):60–62, 1938.

[3] Schott J. *Matrix Analysis for Statistics.* John Wiley and Sons, 1997.

[4] Chen J and Chen Z. Extended bayesian information criteria for model selection with large model spaces. *Biometrica*, 95:759–771, 2008.

[5] Prashant Rangarajan, Sandeep Mody, and Madhav Marathe. Forecasting dengue and influenza incidences using a sparse representation of google trends, electronic health records, and time series data. *PLOS Computational Biology*, 15:e1007518, 11 2019.

[6] Ding M, Chen Y, and Bressler S L. Granger causality: Basic theory and application to neuroscience. In B Schelter, M Winterhalder, and J Timmer, editors, *Handbook of Time Series Analysis*, pages 451–474. Wiley-VCH Verlage, 2006.

[7] Geweke J. Measurement of linear dependence and feedback between multiple time series. *Journal of the American Statistical Association*, 77(378):304–313, 1982.

[8] Wen X, Rangarajan G, and Ding M. Multivariate granger causality: an estimation framework based on factorization of the spectral density matrix. *Philosophical Transactions of the Royal Society of London A: Mathematical, Physical and Engineering Sciences*, 371(1997):20110610, 2013.

[9] Chen Y, Bressler S L, and Ding M. Frequency decomposition of conditional granger causality and application to multivariate neural field potential data. *Journal of Neuroscience Methods*, 150:228–237, 2006.

[10] Geweke J. Measures of conditional linear dependence and feedback between time series. *Journal of the American Statistical Association*, 79:907–915, 1984.

[11] Li T, Weng C Y, King wah E, and Lin C W. Large-scale Stein and Lyapunov equations, Smith method, and applications. *Numerical Algorithms*, 63:727–752, 2013.

[12] Arnaud Delorme and Scott Makeig. Eeglab: an open source toolbox for analysis of single-trial eeg dynamics. *Journal of Neuroscience Methods*, 134:9–12, 01 2004.

[13] Sander Koelstra, Christian Mühl, Mohammad Soleymani, Jong-Seok Lee, Ashkan Yazdani, Touradj Ebrahimi, Thierry Pun, Anton Nijholt, and Ioannis Patras. Deap: A database for emotion analysis using physiological signals. *IEEE Transactions on Affective Computing*, 3:18–31, 12 2011.
